# Supplementary material for: Halo-fluorescein for photodynamic bacteria inactivation in extremely acidic conditions
Source: Nat Commun. 2021 Jan 22;12:526. doi: 10.1038/s41467-020-20869-8 (PMC7822816; doi:10.1038/s41467-020-20869-8)
Supplement: Supplementary file 1 — Supplementary Information [file 41467_2020_20869_MOESM1_ESM.pdf]

Supplementary Information

Halo-Fluorescein for Photodynamic Bacteria Inactivation in Extremely Acidic Conditions

Ying Wang,[1,2] Jiazhuo Li,[1,2] Zhiwei Zhou,[3] Ronghui Zhou,[4] Qun Sun,[3] Peng Wu[1,2]*

[1]State Key Laboratory of Hydraulics and Mountain River Engineering, Sichuan University, Chengdu, 610064, China.

[2]Analytical & Testing Center, Sichuan University, Chengdu, 610064, China.

[3]College of Life Science, Sichuan University, Chengdu, 610064, China.

[4]State Key Laboratory of Oral Diseases, West China Hospital of Stomatology, Sichuan University, Chengdu, 610041, China.

*corresponding author' E-mail: wupeng@scu.edu.cn

Table of Contents

Section S1. Experimental Section 3
S1.1 Materials 3
S1.2 Apparatus 4
Section S2. Research Background 5
S2.1 Common antibacterial agents in extremely acidic condition 5
S2.2 pH dependence of fluorescein equilibria[2] 6
Section S3. Synthesis of 2, 4, 5, 7 – tetrachlorofluorescein 7
Section S4. Theoretical Calculations 9
S4.1 Calculation for frontier orbitals 9
S4.2 Calculation for ESP maps 11
Section S5. pH-dependent Properties of the Halogenated Fluoresceins. 12
S5.1 pH titration of halogenated fluorescein 12
S5.2 Singlet oxygen phosphorescence emission under different pH 23
S5.3 $^1O_2$ phosphorescence emission of TIF under different pH and irradiance 24
Section S6. Photophysical Characterizations 25
S6.1 Absorption, fluorescence, and phosphorescence spectra 25
S6.2 Fluorescence lifetime 29
S6.3 Relative singlet oxygen quantum yield 29
S6.4 Transient absorption spectra 36
S6.5 Photophysical properties of the halogenated-fluorescein derivatives 38
Section S7. Basic Performances of TIF in Photodynamic Bacteria Inactivation 39
S7.1 Bacteria inactivation performance of different photosensitizers 39
S7.2 International standards and limits of TIF 39
S7.3 *Lactobacillus* inactivation performance of TIF at different pH 40
S7.4 Identification of specific ROS generated from TIF 40
S7.5 The surface charge of E. coli in different solvents 42
Section S8. Inactivation performance of TIF for different bacterial strains 43
Section S9. Photodynamic Antibacterial Inactivation Performance of TIF in Acidic Juices 49
S9.1 Biocompatibility of TIF 49
S9.2 The antimicrobial activity of TIF under different LED irradiation 50
S9.3 Photodynamic antibacterial in juices 51
S9.4 Long-term colonies numbers monitoring 54
S9.5 Antioxidants contents in juices 54
S9.6 Photodynamic antibacterial with TIF for fresh Fruit preservation 58
Section S10. In vivo photodynamic antimicrobial chemotherapy of oral Candidiasis. 59

Section S1. Experimental Section

S1.1 Materials

2,4-Dihydroxybenzoic acid, N,N-Dimethylaniline, phthalic anhydride, tetrachlorophthalic anhydride, phthalic anhydride, methanesulfonic acid were purchased from Aladdin (Shanghai, China) without further purification. Hydrochloric acid, hydrogen peroxide, acetic acid, methanol and acetonitrile were purchased from Kelong Reagent Co. (Chengdu, China). The information about the photosensitizers were given in Supplementary Table 1 below.

Supplementary Table 1. The information of halo-fluorescein in this work.

Abbr.	Name	CAS	Source	Price (USD)
FL	Fluorescein	2321-07-5	Yuanye	1.6 / 5 g
DCF	4’,5’-dichlorofluorescein	2320-96-9	Yuanye	3.3 / 5 g
DBF	4’,5’-dibromofluorescein	596-03-2	J&K	25.1 / 5 g
DIF	4’,5’-diiodofluorescein	33239-19-9	Alfa-Aesar	18.2 / 5 g
TCF-1	2’,4’,5’,7’-tetrachlorofluorescein	-	-	-
EY	2’,4’,5’,7’-tetrabromofluorescein	17372-87-1	Aladdin	9.7 / 5 g
TIF	2’,4’,5’,7’-tetraiodofluorescein	16423-68-0	Aladdin	16.6 / 5 g
TCF-2	3,4,5,6- tetrachlorofluorescein	6262-21-1	TCI	153.2 / 5 g
OCF	2’,4’,5’,7’-tetrachloro-3,4,5,6-tetrachlorofluorescein	-	-	-
PB	2',4',5',7'-tetrabromo-3,4,5,6-tetrachlorofluorescein	18472-87-2	Aladdin	3.2 / 5 g
RB	2',4',5',7'-tetrabromo-3,4,5,6-tetraiodofluorescein	632-69-9	Aladdin	53.9 / 5 g
TCF-1 and OCF was synthesized according to ref. 1, see Section 4 of the SI.				

S1.2 Apparatus

All the instrumental information used for characterizations were listed in Supplementary Table 2.

Supplementary Table 2. The instrumental information used for characterizations in this work.

Characterization items	Type	Manufacturer
^1H NMR	Avance II-400 MHz	Bruker, Switzerland
UV/Vis absorption spectra	Lambda-365 spectormeter	Perkin Elmer, USA
Fluorescence titration	F-7000 spectrofluorometer	Hitachi, Japan
Fluorescence and phosphorescence spectra	FluoroMax-4P spectrofluorometer	Horiba Scientific, USA
Fluorescence lifetime & QY & $^1O_2$ emission	Fluolog-3 spectrofluorometer with an integration sphere (IS80, Labsphere)<br>Fluorescence lifetime excitation: DeltaDiode (505 nm)	Horiba Jobin Yvon, USA
EPR	Bruker EMX plus	Bruker, Switzerland
Transient absorption spectra	EOS	Ultrafast Systems, USA
Cell imaging	Nikon A1+	Nikon, Japan
Zeta potential	Zetasizer Nano ZS	Malvern, England

Section S2. Research Background

S2.1 Common antibacterial agents in extremely acidic condition

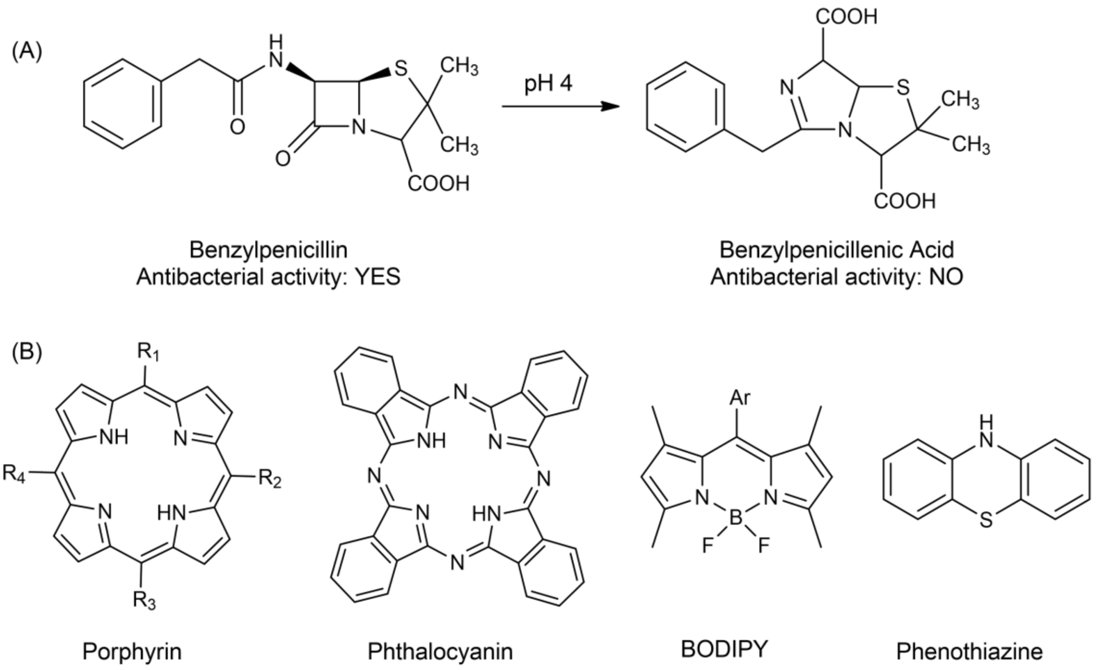

Supplementary Figure 1. (A) Structural change of penicillin in extremely acidic condition. (B) The structures of several photosensitizers commonly used in PDT and photodynamic inactivation.

S2.2 pH dependence of fluorescein equilibria[2]

Upon protonation, fluorescein experience changes as follows. Structure I is the most stable colored and fluorescent form, whereas Structure V is the most stable colorless and non-fluorescent.

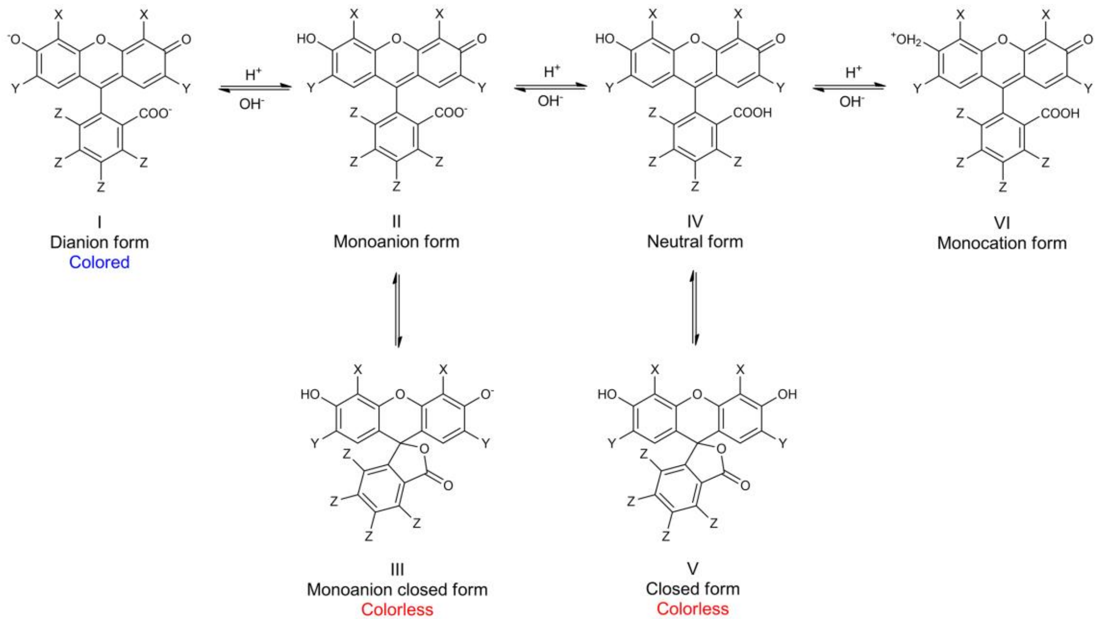

Supplementary Figure 2. pH-dependence of fluorescein equilibrium.

Section S3. Synthesis of 2, 4, 5, 7 – tetrachlorofluorescein

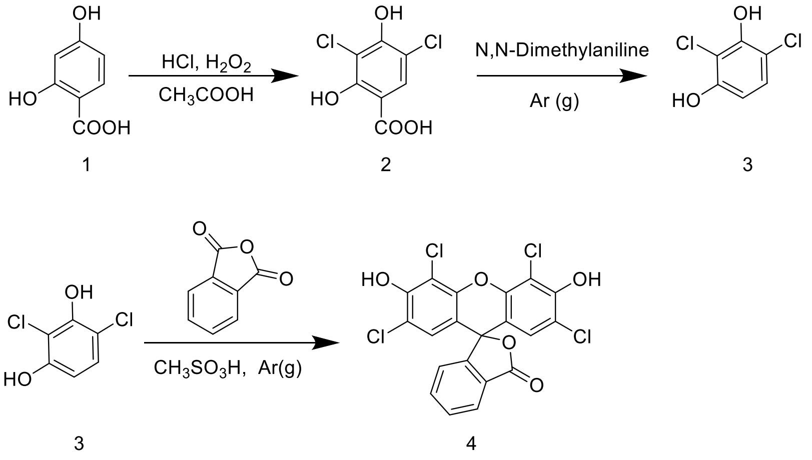

Supplementary Figure 3. Synthesis procedures of TCF-1.

Compound 2 and 3 were synthesized followed by Ref 1. 2,4-dichlororesorcinol 3 (5.25 mmol) and o-phthalic anhydride (2.48 mmol) were dissolved in methanesulfonic acid and the mixture were heated to 150 °C under Ar (g). Then, the reaction mixture poured into ice-water. The precipitated solid were collected by suction filtration and washed with ice-water. The dark pink solid was purified by silica gel chromatograph to get target compound 4. ^1H NMR (DMSO, 400 MHz), δ: 10.61 (s, 2H, Ar-OH); 8.20 (s, H, 3'-ArH); 7.69 (s, 2H, 4', 5'-ArH); 7.34 (s, H, 6'-ArH); 6.74 (s, 2H, 1,8-ArH).

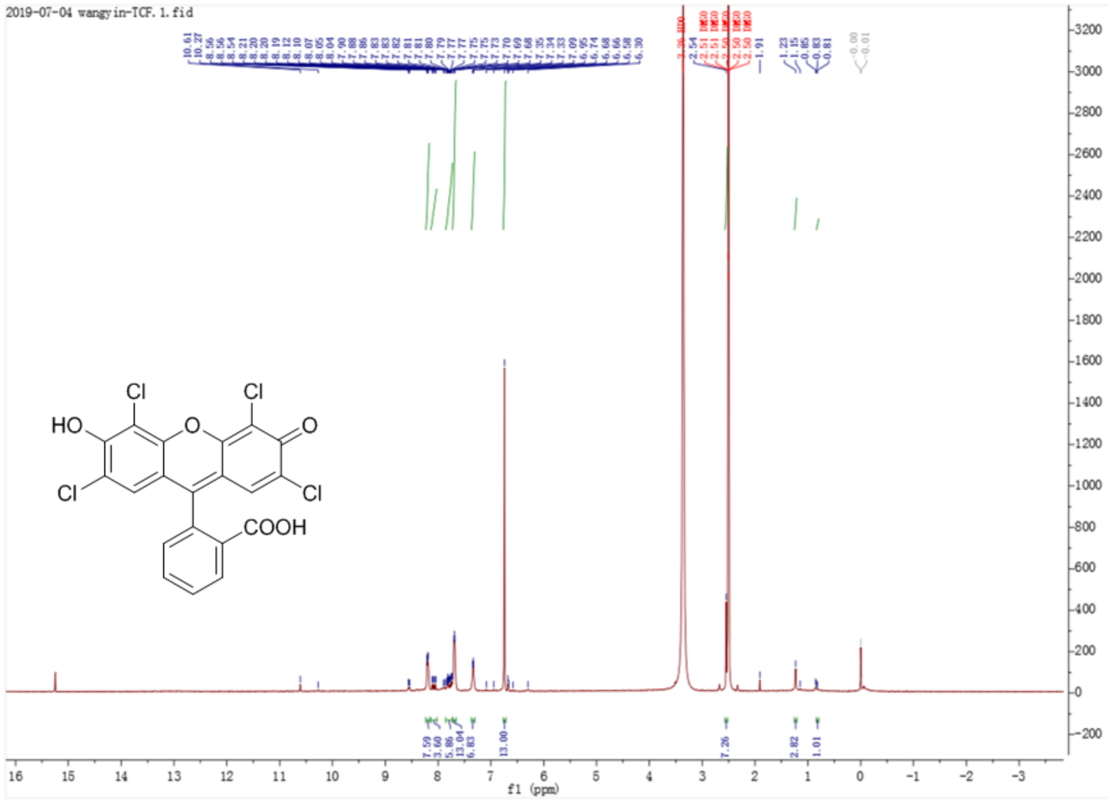

Supplementary Figure 4. ^{1}H NMR of **TCF-1**.

Section S4. Theoretical Calculations

S4.1 Calculation for frontier orbitals

Structure optimization and charge analysis for ground state open form of halogenated fluorescein derivatives were calculated at B3LYP level with Gaussian 09. The 6-31G** basis were set for C, H, O, and Cl, and the LANL2dz basis were set for Br and I. Visualization of HOMO and LUMO were performed with Gaussian View.

Supplementary Table 3. Chemical structures and HOMO/LUMO of the photosensitizers used in this work (B3LYP/6-31G** level with Gaussian 09).

	Structure	HOMO	LUMO
FL	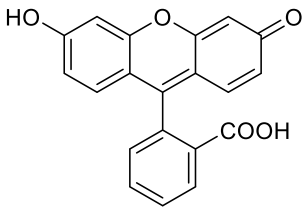	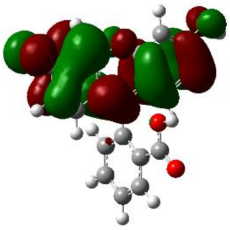	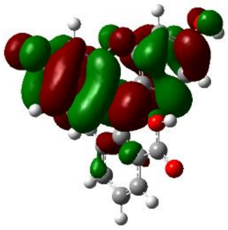
DCF	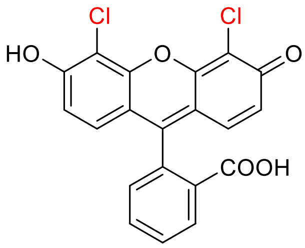	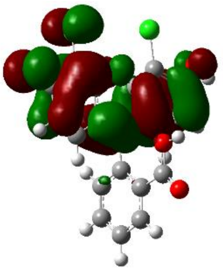	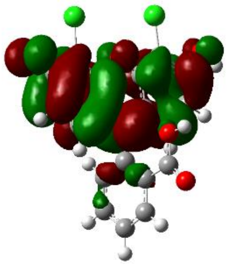
DBF	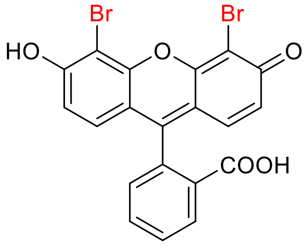	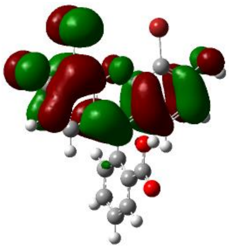	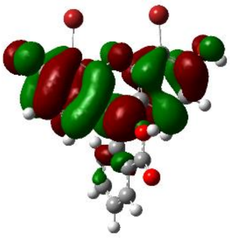
DIF	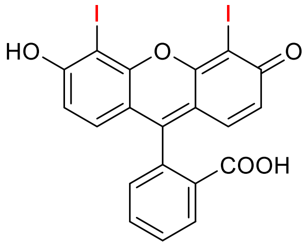	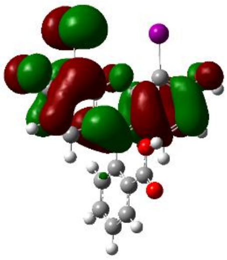	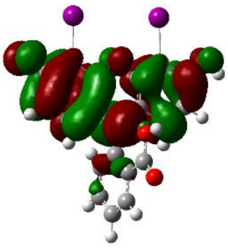

TCF-1	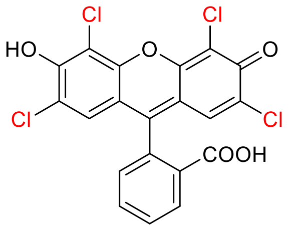	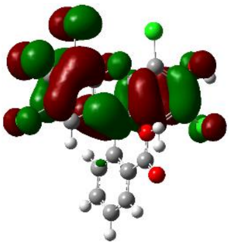	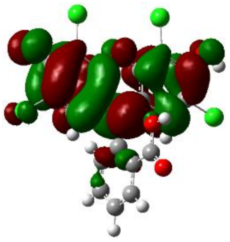
EY	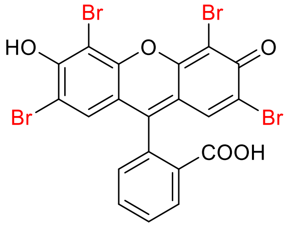	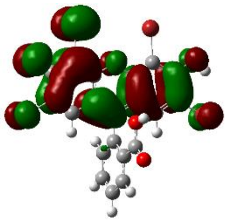	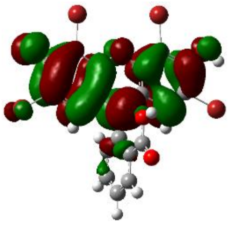
TIF	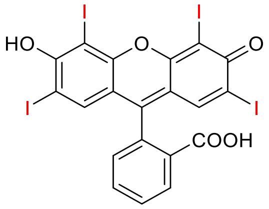	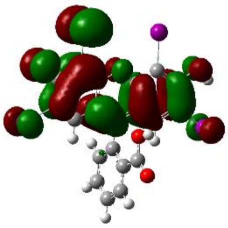	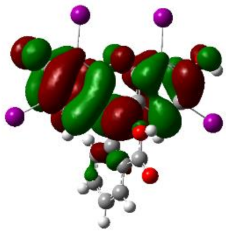
TCF-2	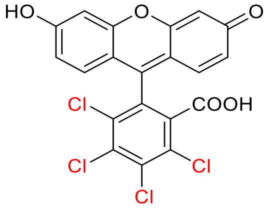	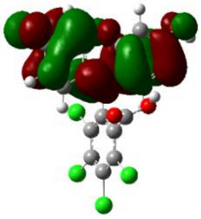	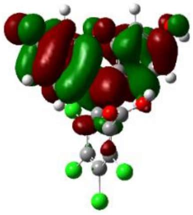
OCF	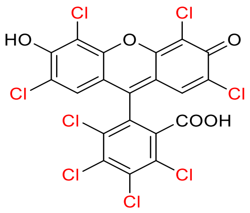	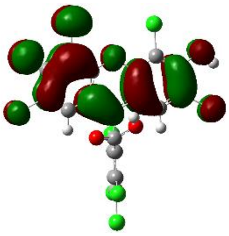	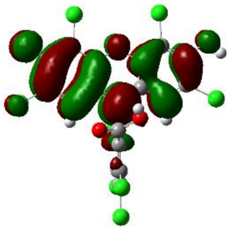
PB	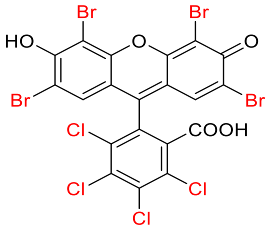	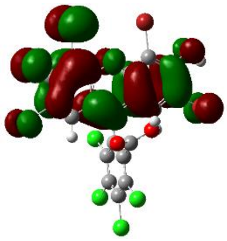	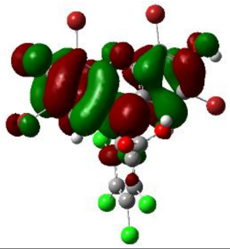
RB	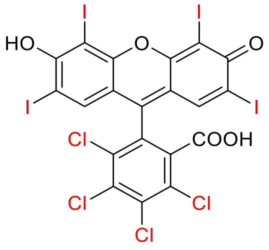	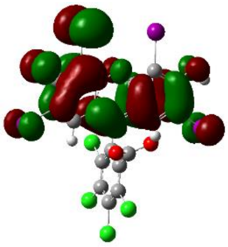	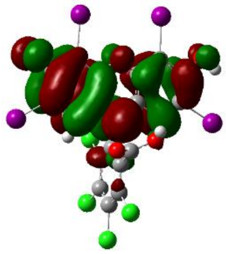

S4.2 Calculation for ESP maps

ESP map for ground state halogenated fluorescein derivatives were used to evaluate the reactivity of spirocyclization reaction. Geometry optimization was calculated at B3LYP/6-31G** basis with Gaussian 09. Quantitative analysis of molecular surface of fluorescein derivatives were calculated at Multiwfn software package.[3, 4] Visualization of ESP map were performed with VMD software package.

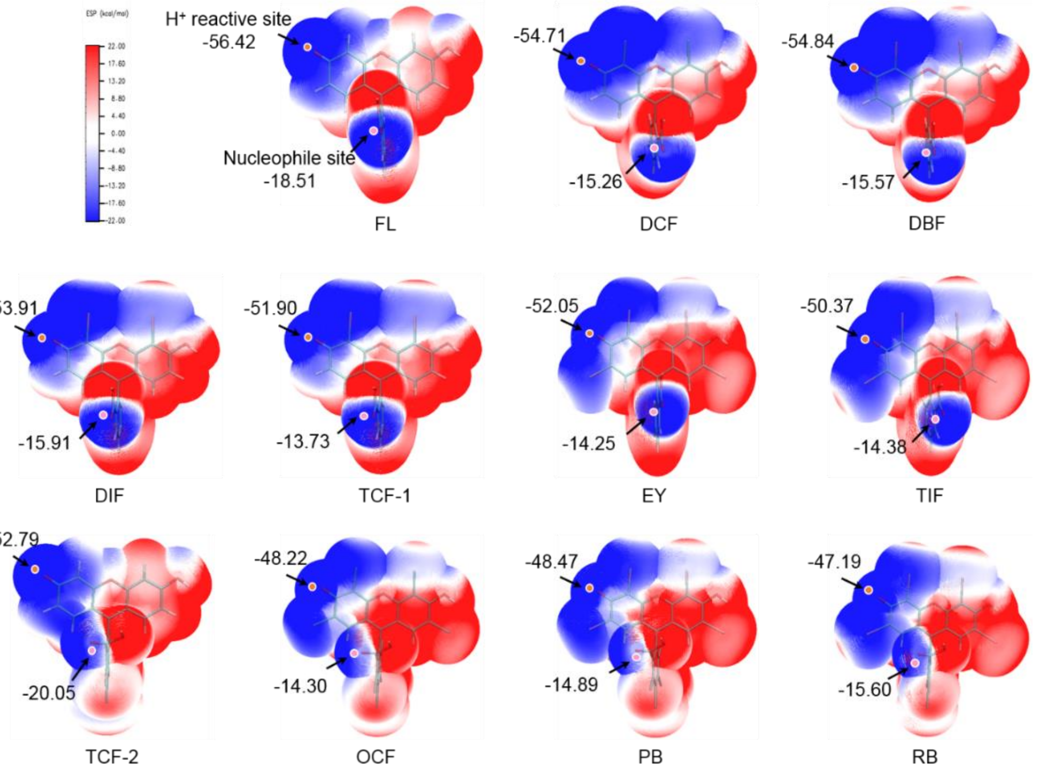

Supplementary Figure 5. ESP map of halogenated fluorescein derivatives.

Section S5. pH-dependent Properties of the Halogenated Fluoresceins.

S5.1 pH titration of halogenated fluorescein

Fluorescein derivatives (Supplementary Table 1) were dissolved in DMSO to obtain 1 mM stock solutions and then diluted with citric acid-Na_2HPO_4 buffer (100 mM) to 10 μM. The pH of the solutions were adjusted through varying the ratios of citric acid and Na_2HPO_4 (pH 2.2-8.0), and further acidity was adjusted with concentrated HCl or 1 M NaOH. The absorption and fluorescence spectra of the resultant solutions were then collected.

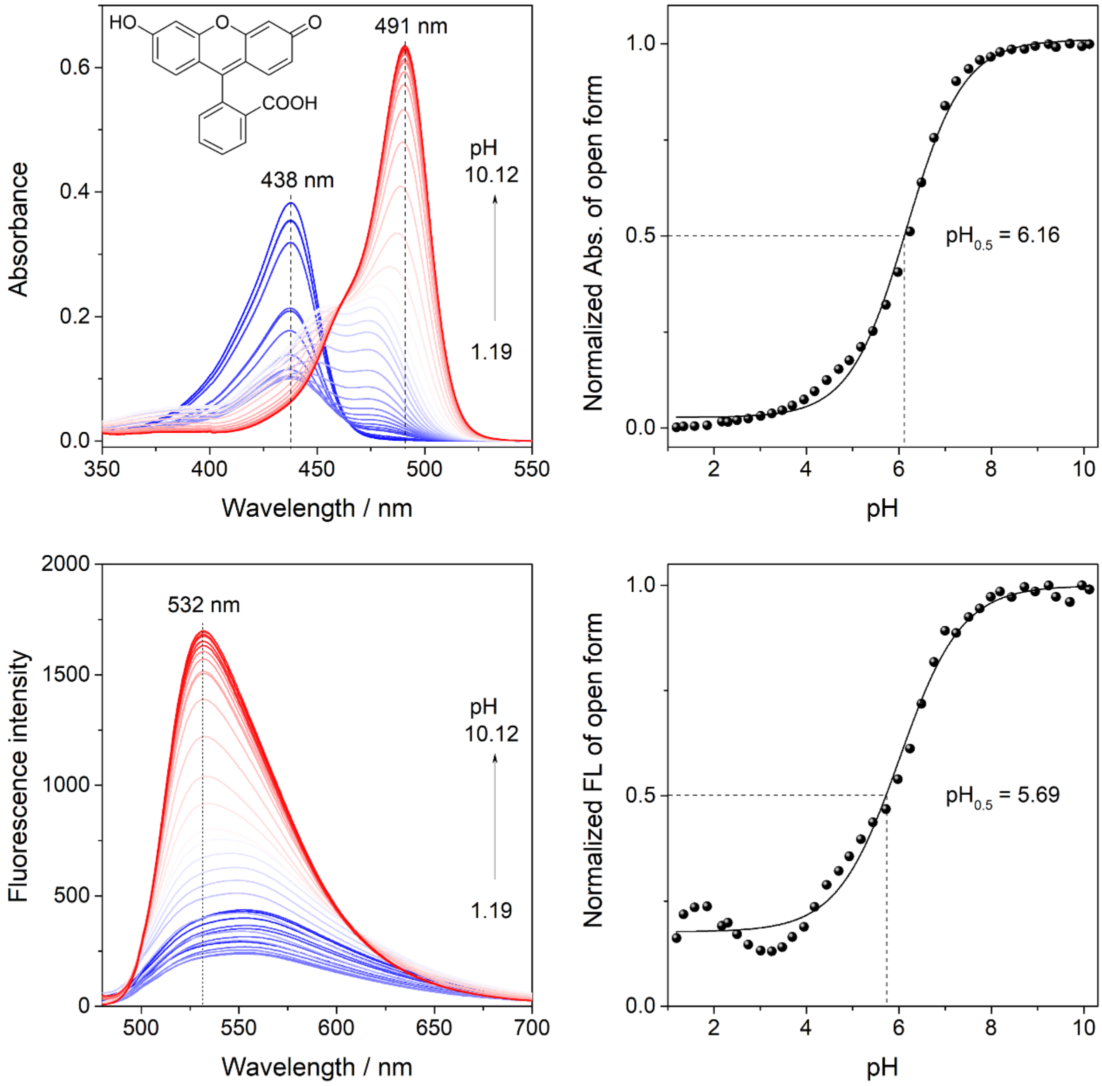

Supplementary Figure 6. The absorption and fluorescence pH titration curves of FL.

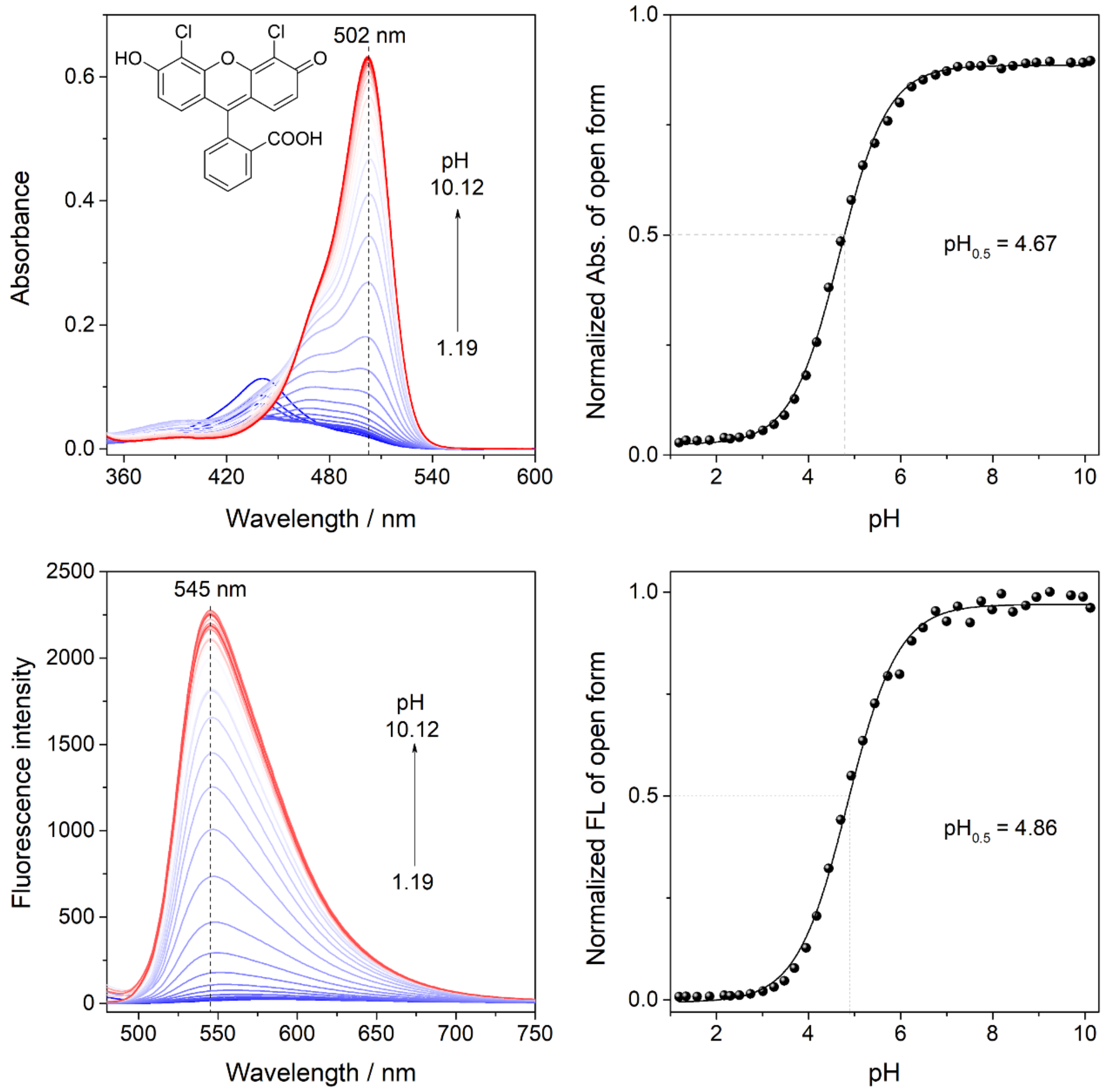

Supplementary Figure 7. The absorption and fluorescence pH titration curves of DCF.

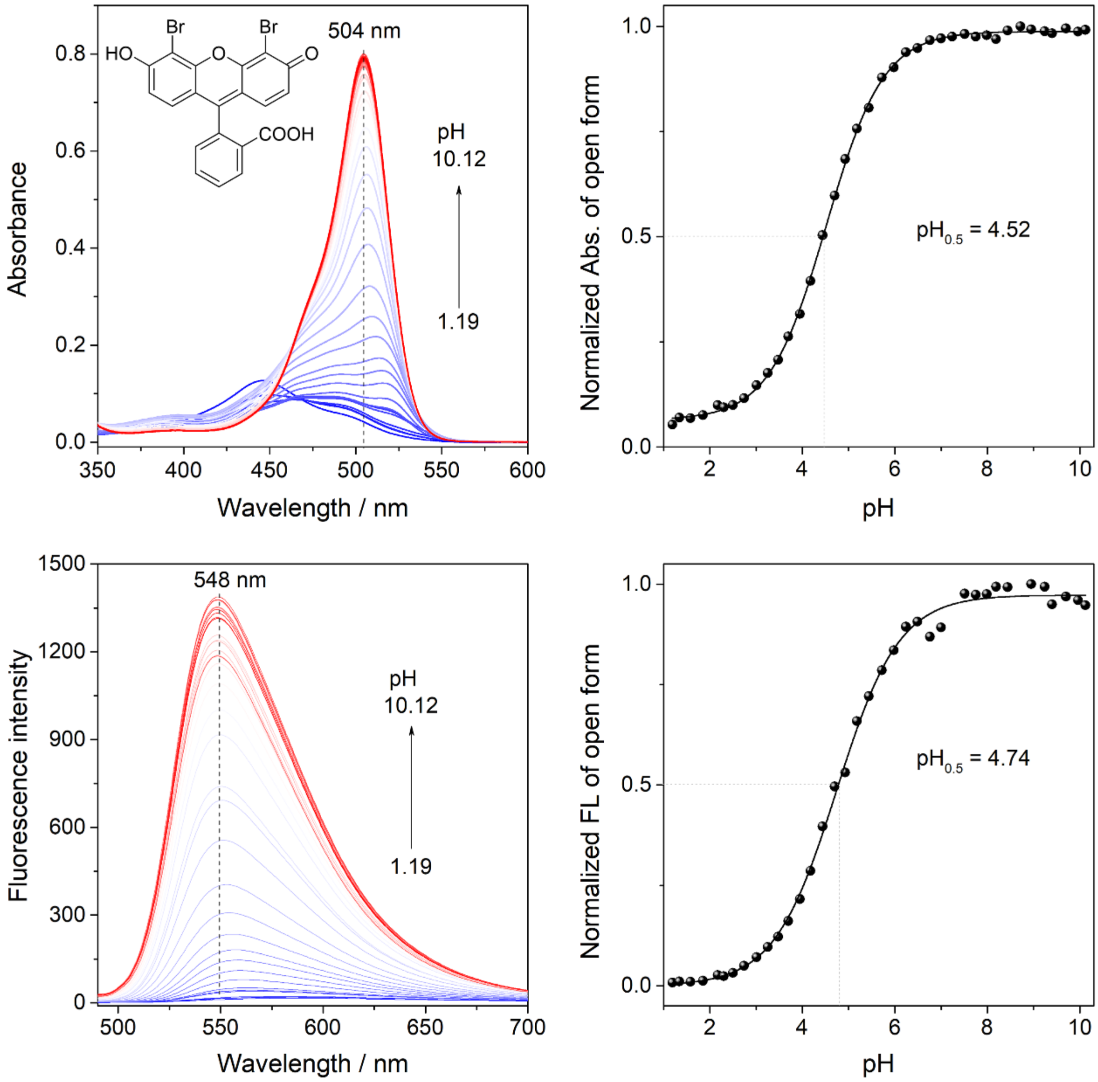

Supplementary Figure 8. The absorption and fluorescence pH titration curves of DBF.

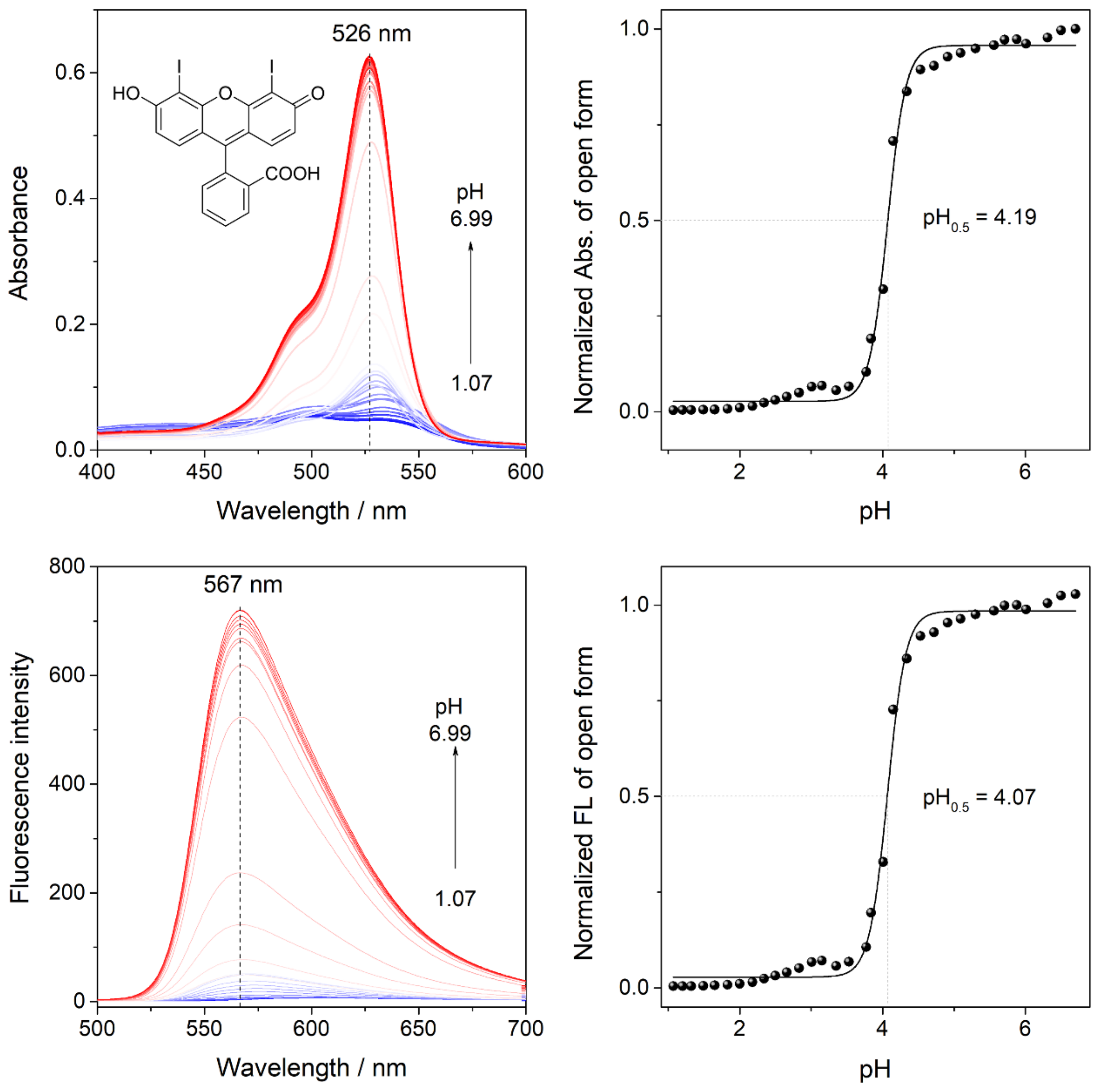

Supplementary Figure 9. The absorption and fluorescence pH titration curves of DIF.

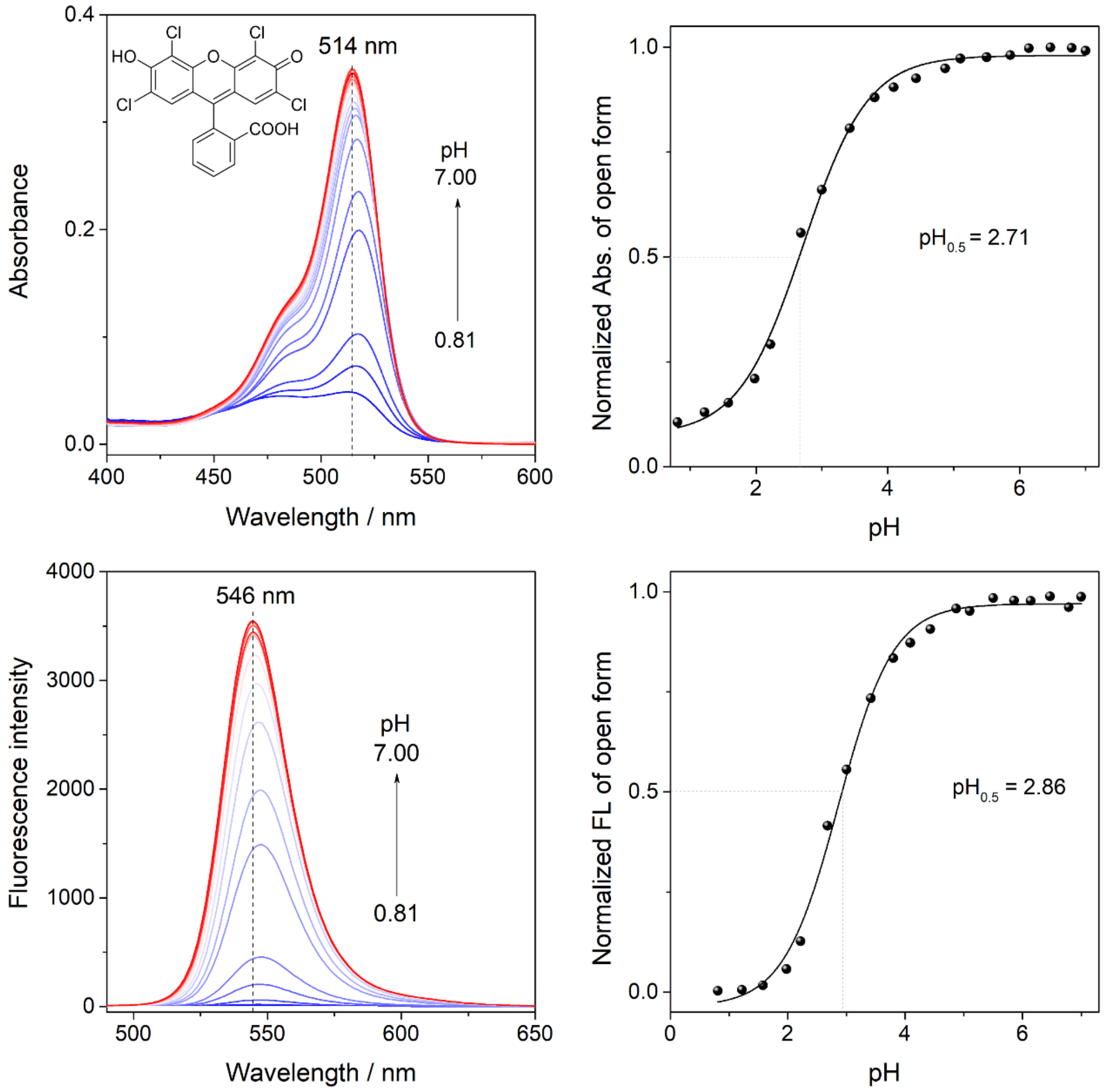

Supplementary Figure 10. The absorption and fluorescence pH titration curves of TCF-1.

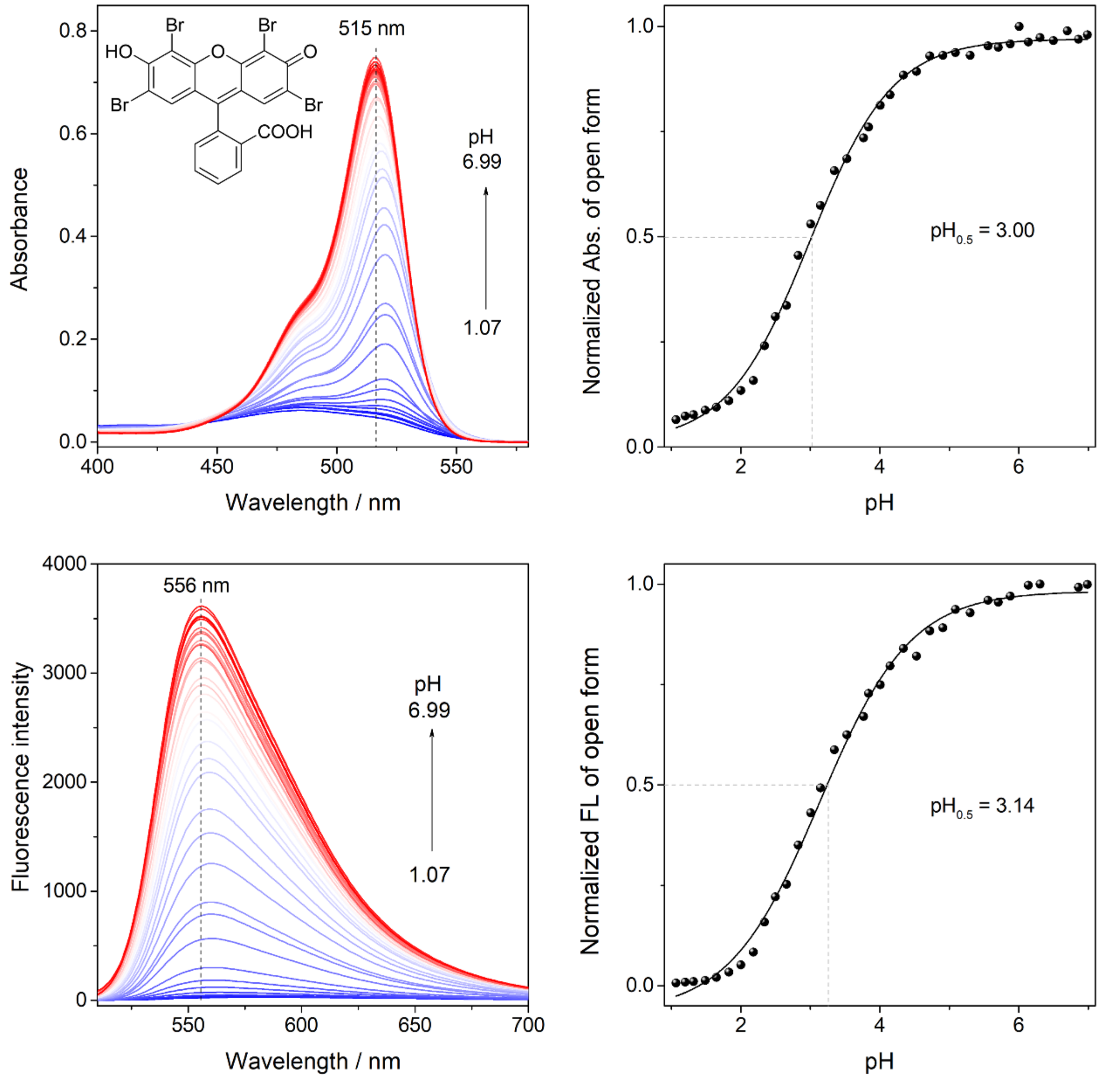

Supplementary Figure 11. The absorption and fluorescence pH titration curves of EY.

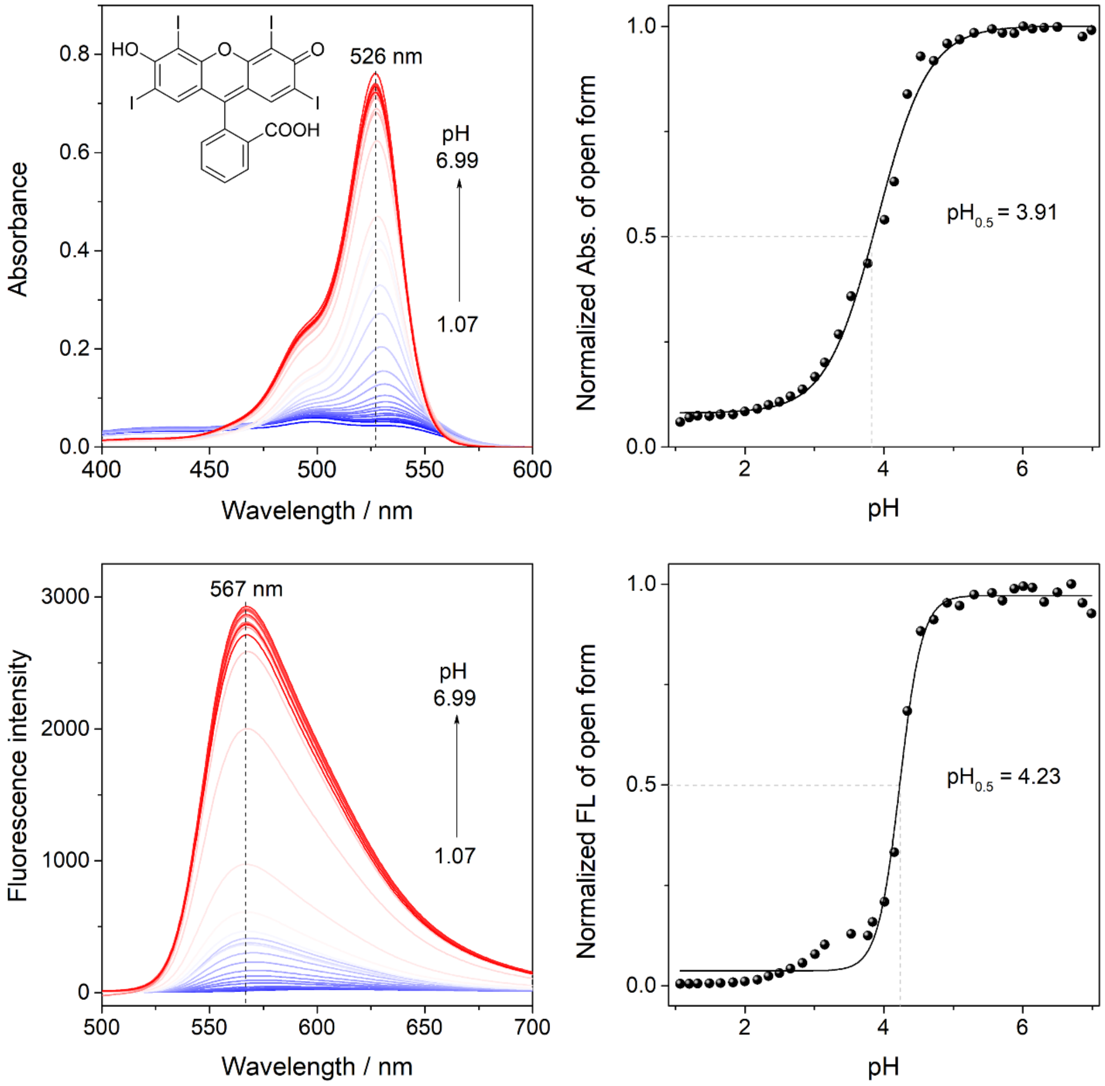

Supplementary Figure 12. The absorption and fluorescence pH titration curves of TIF.

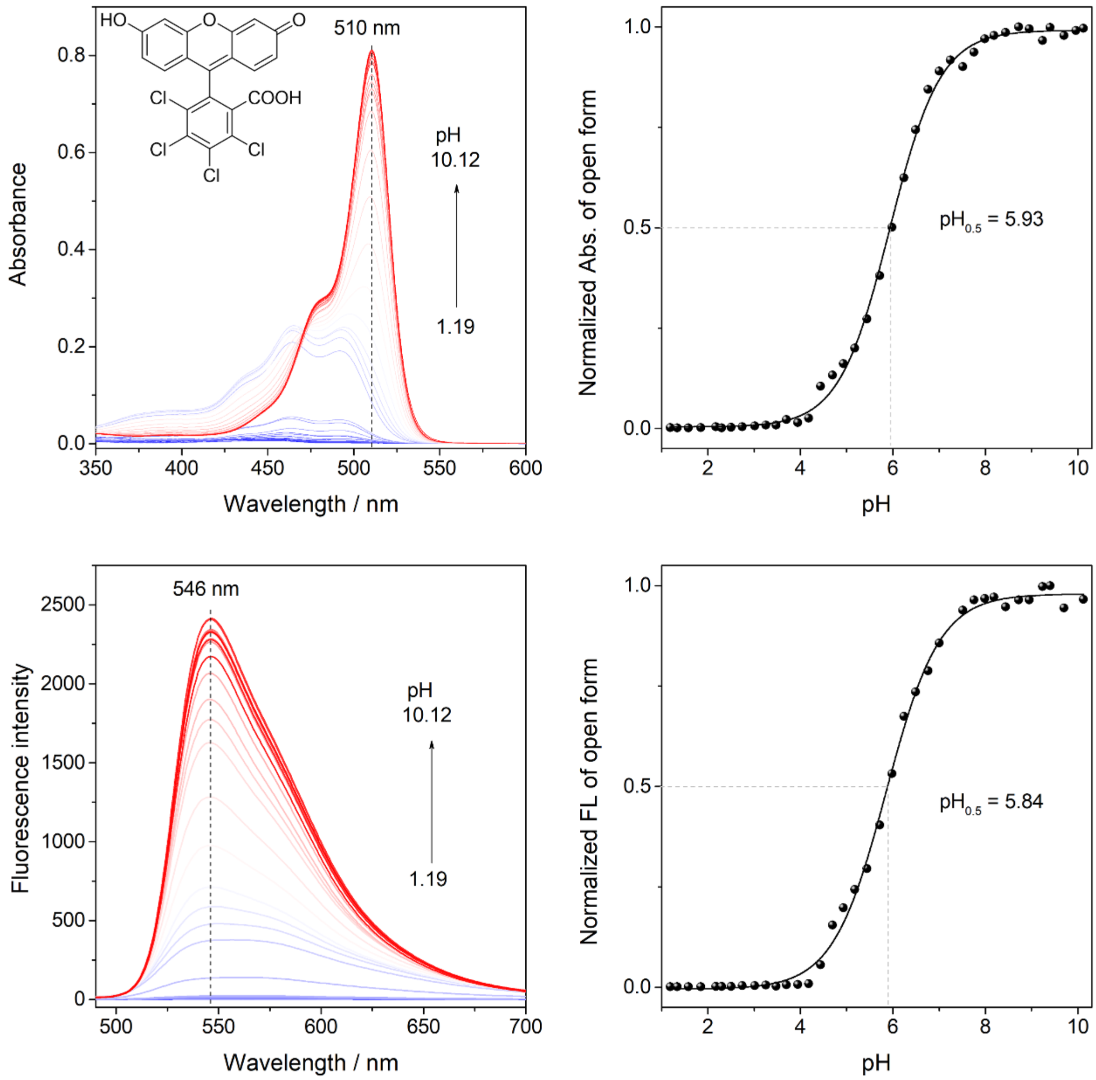

Supplementary Figure 13. The absorption and fluorescence pH titration curves of TCF-2.

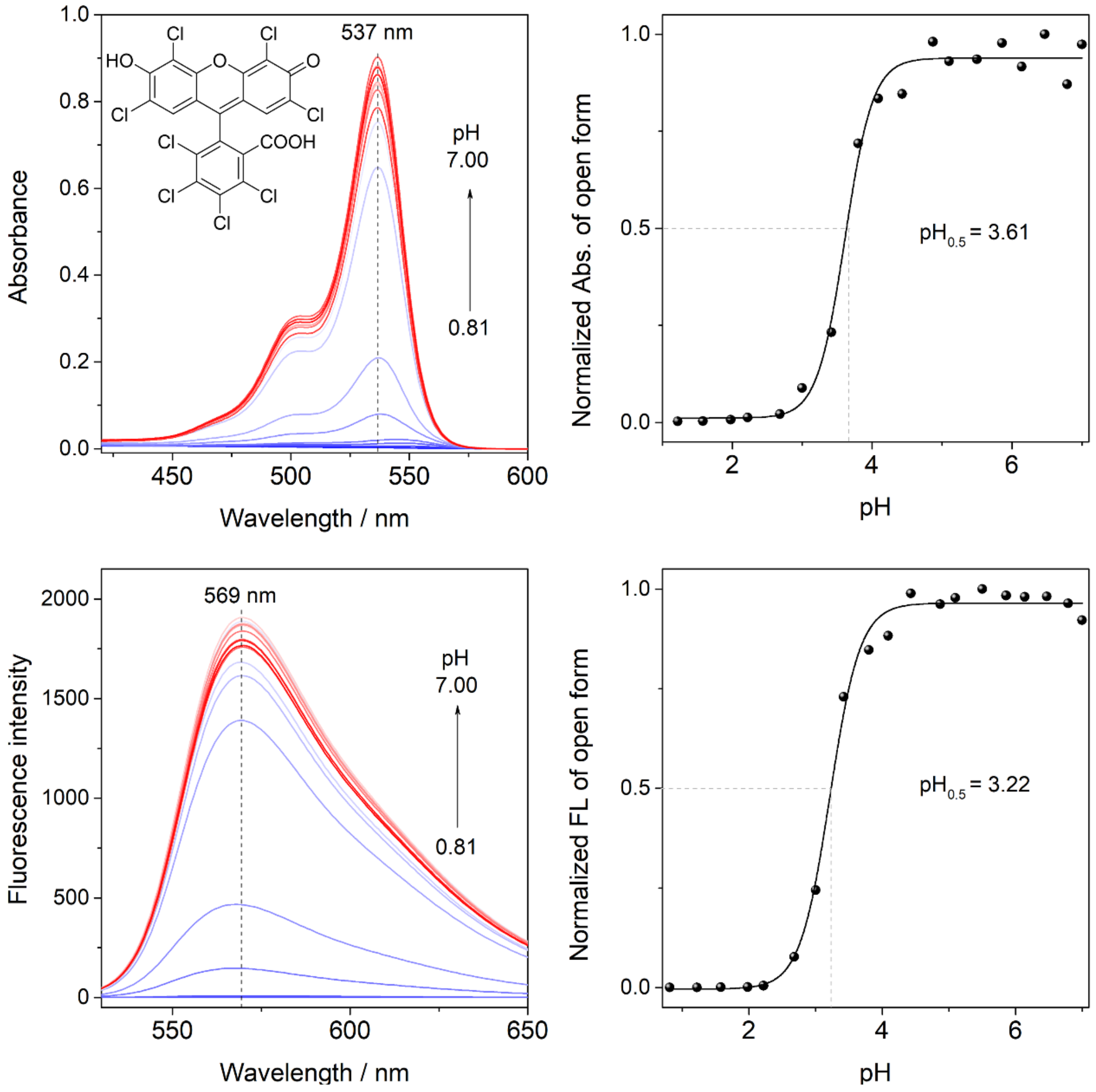

Supplementary Figure 14. The absorption and fluorescence pH titration curves of OCF.

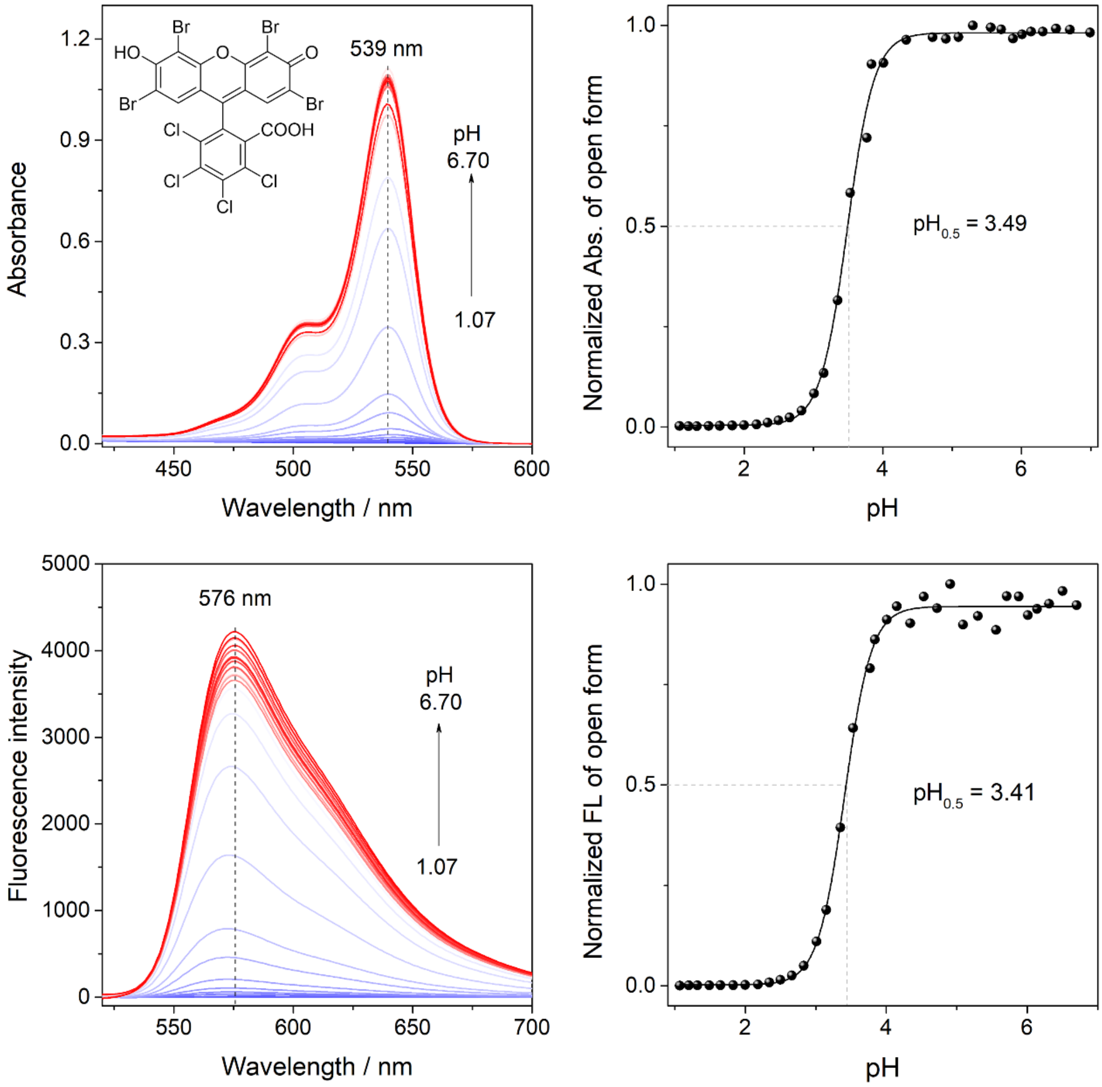

Supplementary Figure 15. The absorption and fluorescence pH titration curves of PB.

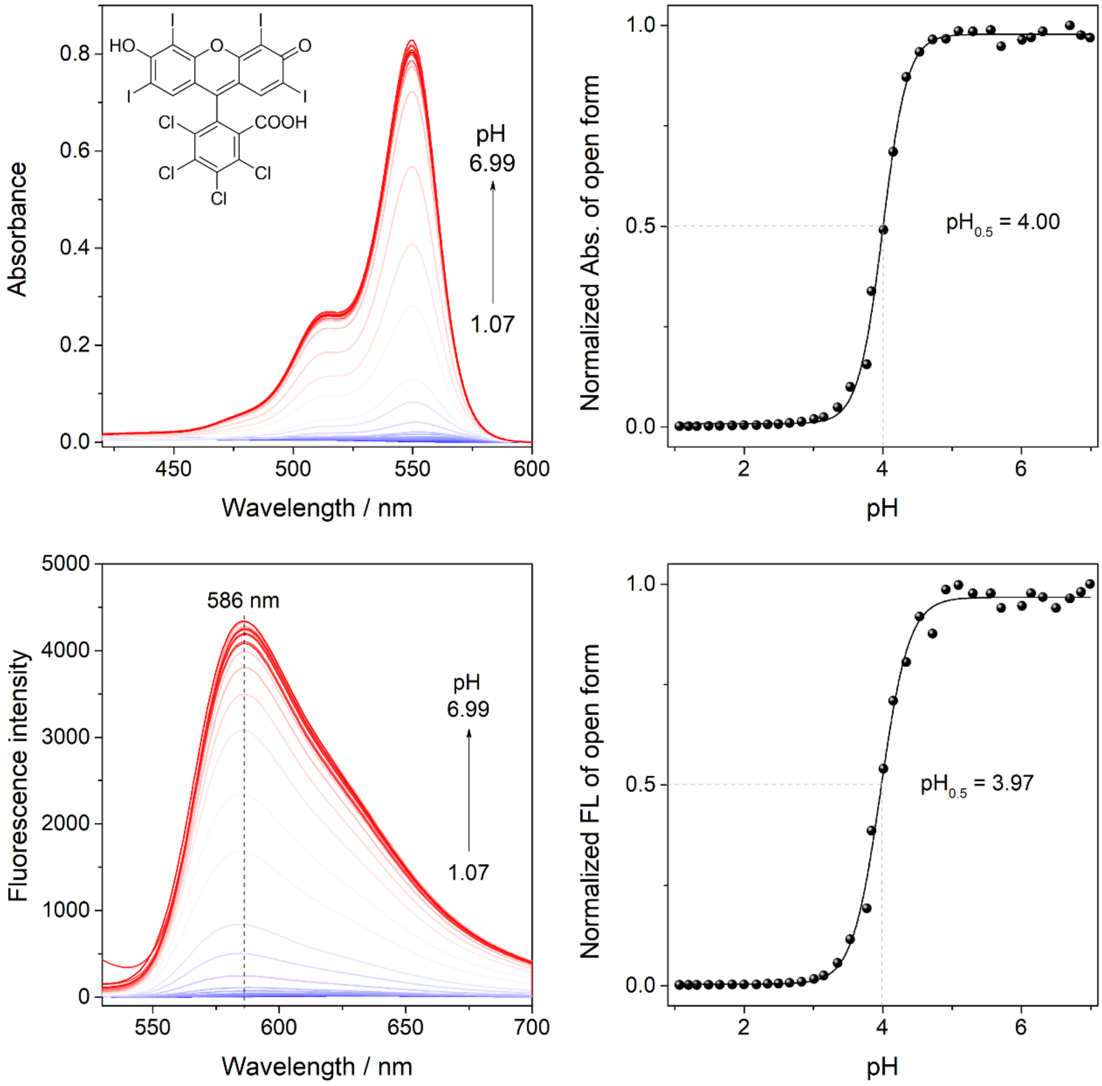

Supplementary Figure 16. The absorption and fluorescence pH titration curves of RB.

S5.2 Singlet oxygen phosphorescence emission under different pH

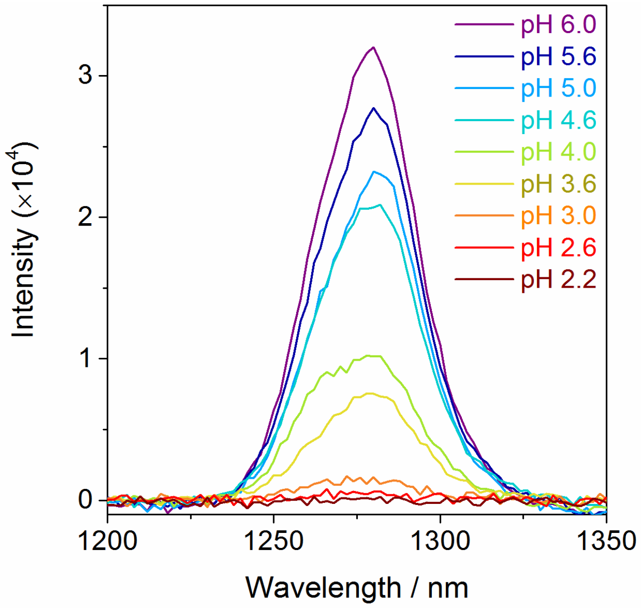

Supplementary Figure 17. The singlet oxygen phosphorescence emission in D_2O under different pH of PB.

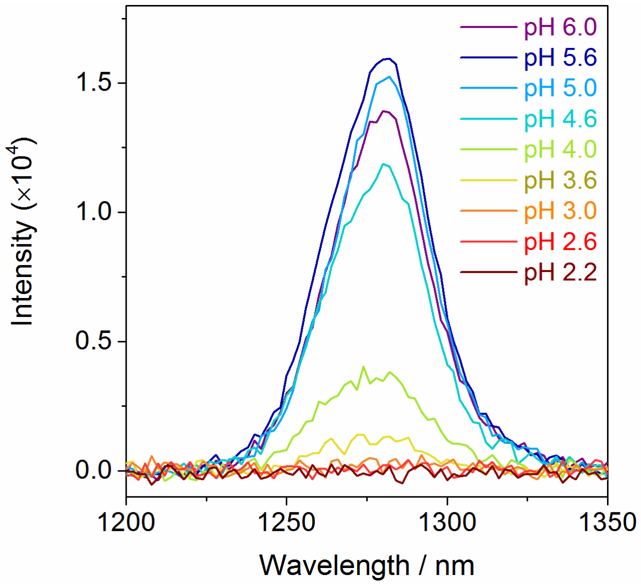

Supplementary Figure 18. The singlet oxygen phosphorescence emission in D_2O under different pH of RB.

S5.3 $^1O_2$ phosphorescence emission of TIF under different pH and irradiance

10 μM TIF solutions were first prepared with citric acid-Na_2HPO_4 buffer (100 mM, diluted by D_2O) to yield different pH (2.2-6.0). The pH value of solution were adjust by HCl (c, diluted by D_2O). Then, laser of 532 nm was selected as the light source, and the $^1O_2$ phosphorescence emission were collected by near infrared detector (H-10330)

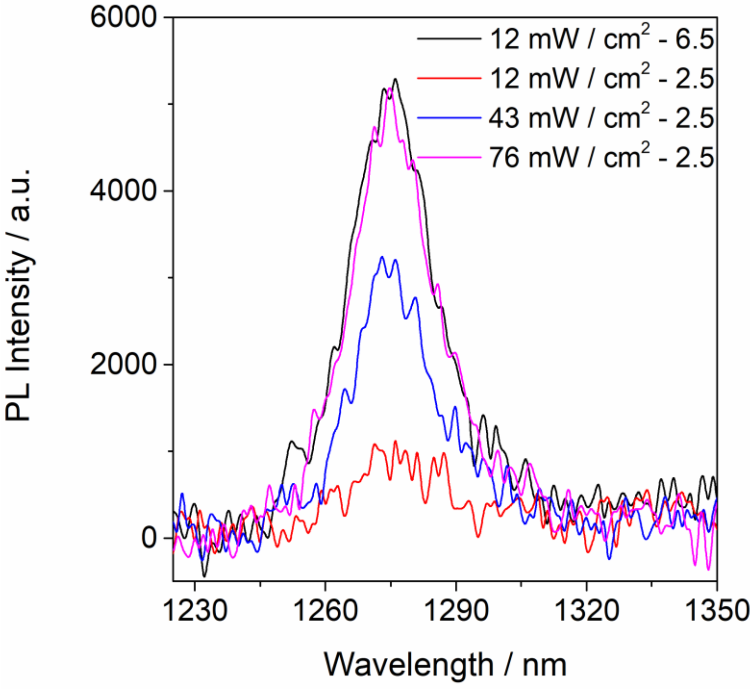

Supplementary Figure 19. The $^1O_2$ phosphorescence emission intensity of TIF under pH 6.5 or pH 2.5, followed by different light irradiance (12 mW/cm^2, 75 mW/cm^2, respectively).

Section S6. Photophysical Characterizations

S6.1 Absorption, fluorescence, and phosphorescence spectra

Fluorescein and its derivatives were dissolved in DMSO to obtain 1 mM stock solutions. The stock solutions were diluted with deionized water to 10 μM.

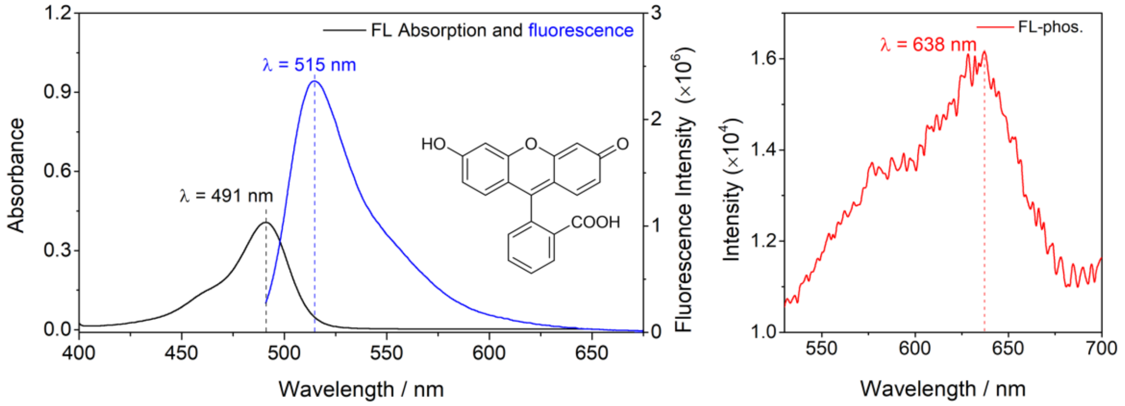

Supplementary Figure 20. The absorption (black), fluorescence (blue), and phosphorescence (red, slit 25 nm) spectra of FL.

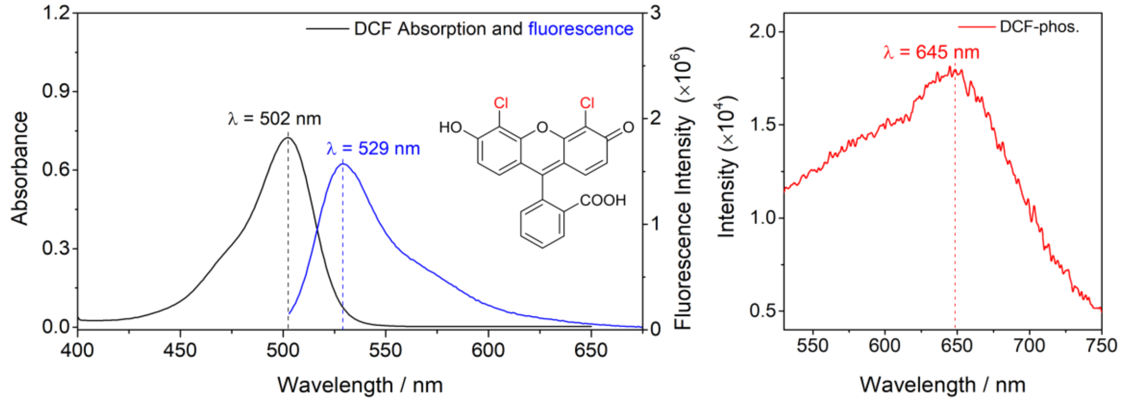

Supplementary Figure 21. The absorption (black), fluorescence (blue), and phosphorescence (red, slit 25 nm) spectra of DCF.

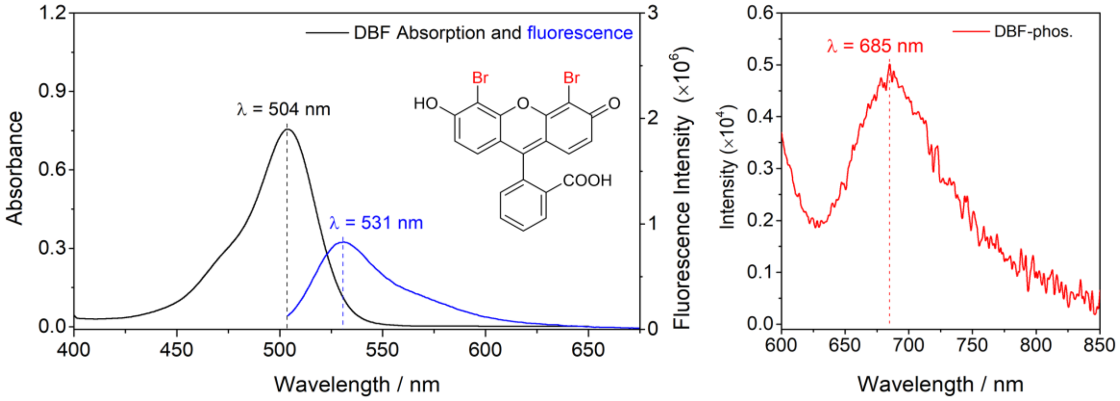

Supplementary Figure 22. The absorption (black), fluorescence (blue), and phosphorescence (red, slit 10 nm) spectra of DBF.

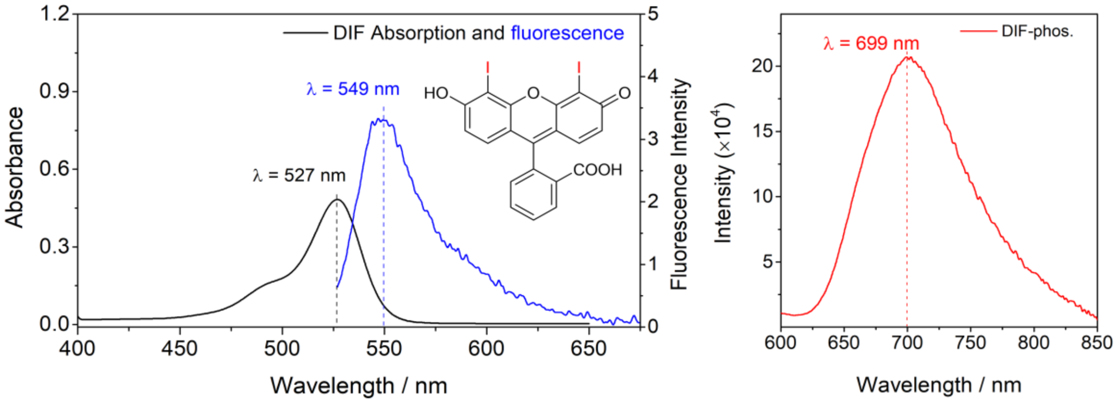

Supplementary Figure 23. The absorption (black), fluorescence (blue), and phosphorescence (red, slit 10 nm) spectra of DIF.

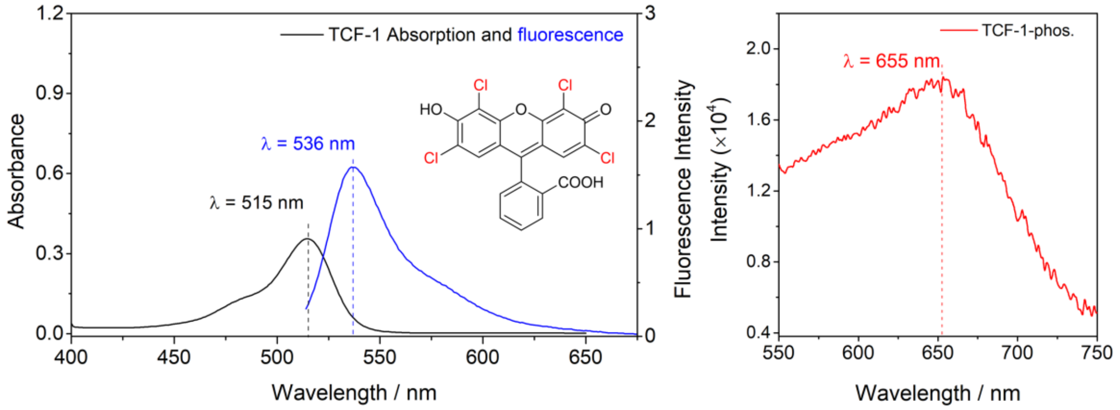

Supplementary Figure 24. The absorption (black), fluorescence (blue), and phosphorescence (red, slit 25 nm) spectra of TCF-1.

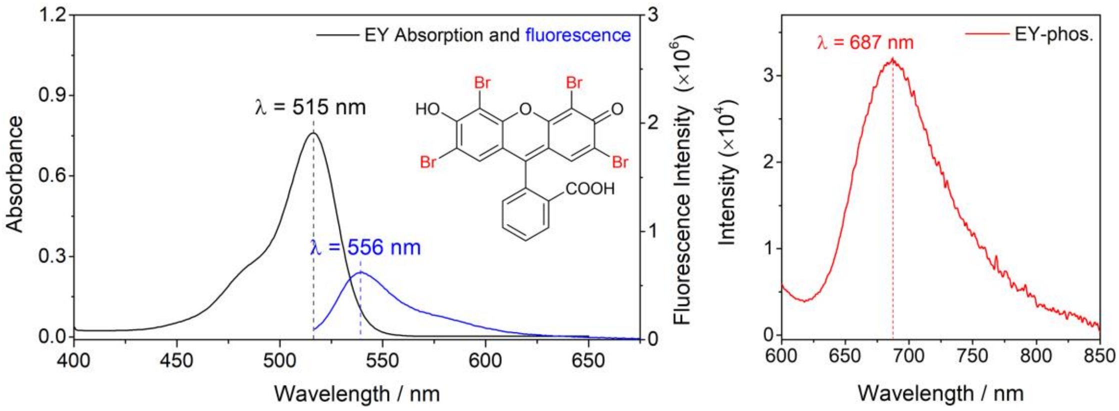

Supplementary Figure 25. The absorption (black), fluorescence (blue), and phosphorescence (red, slit 10 nm) spectra of EY.

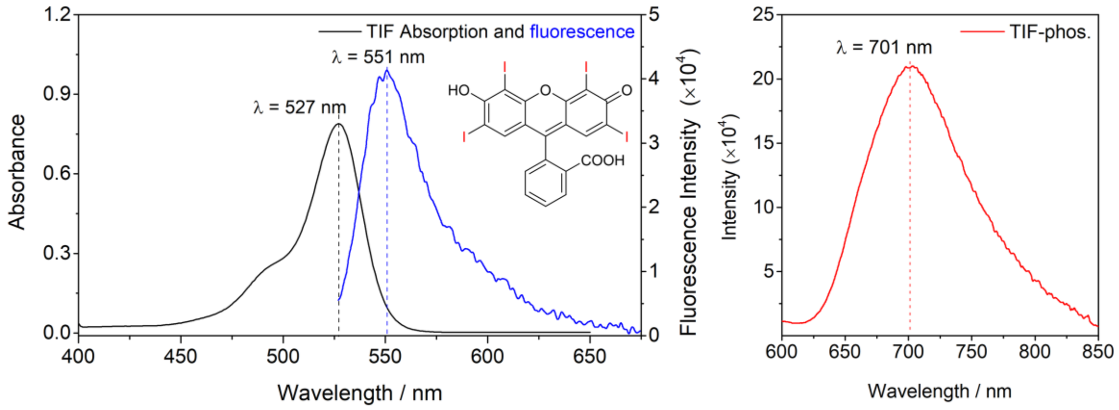

Supplementary Figure 26. The absorption (black), fluorescence (blue), and phosphorescence (red, slit 10 nm) spectra of TIF.

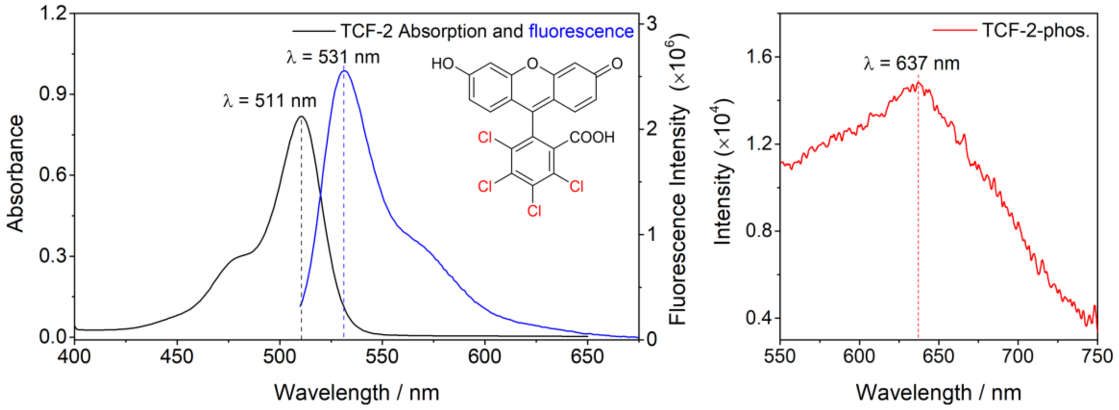

Supplementary Figure 27. The absorption (black), fluorescence (blue), and phosphorescence (red, slit 25 nm) spectra of TCF-2.

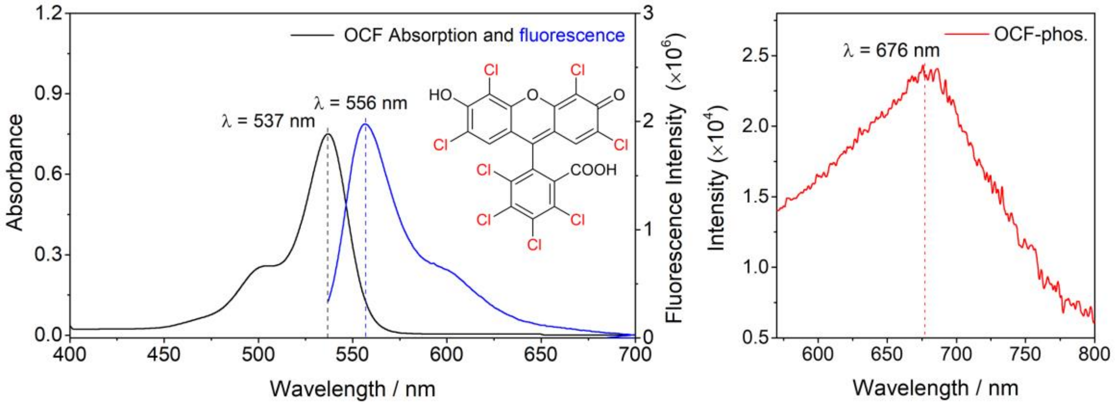

Supplementary Figure 28. The absorption (black), fluorescence (blue), and phosphorescence (red, slit 25 nm) spectra of OCF.

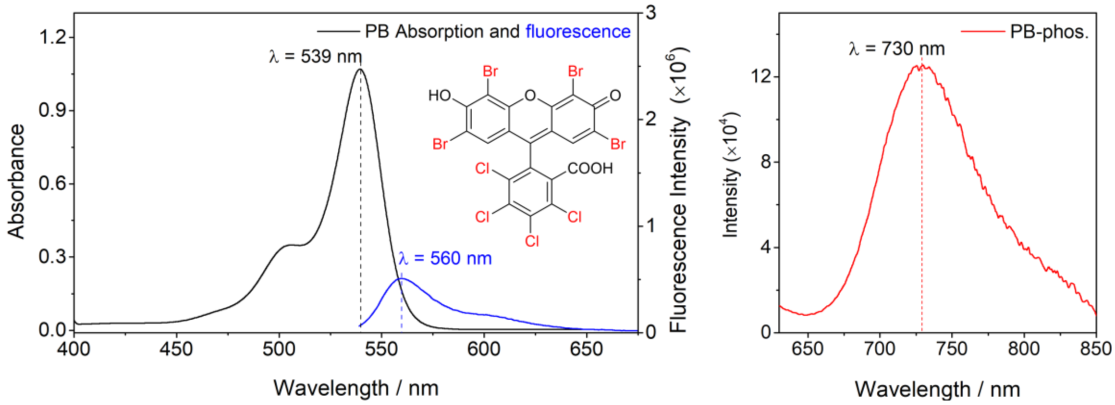

Supplementary Figure 29. The absorption (black), fluorescence (blue), and phosphorescence (red, slit 10 nm) spectra of PB.

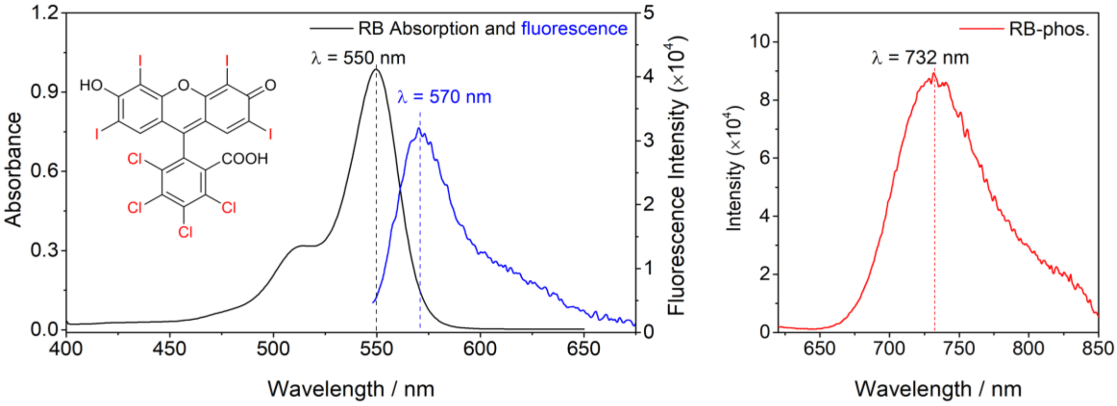

Supplementary Figure 30. The absorption (black), fluorescence (blue), and phosphorescence (red, slit 10 nm) spectra of RB.

S6.2 Fluorescence lifetime

Fluorescence decay samples was recorded with Fluorolog-3 (Horiba). The solution of samples were prepared to be 5 μM in 0.5% (v/v) DMSO, and diluted with 0.1M NaOH.

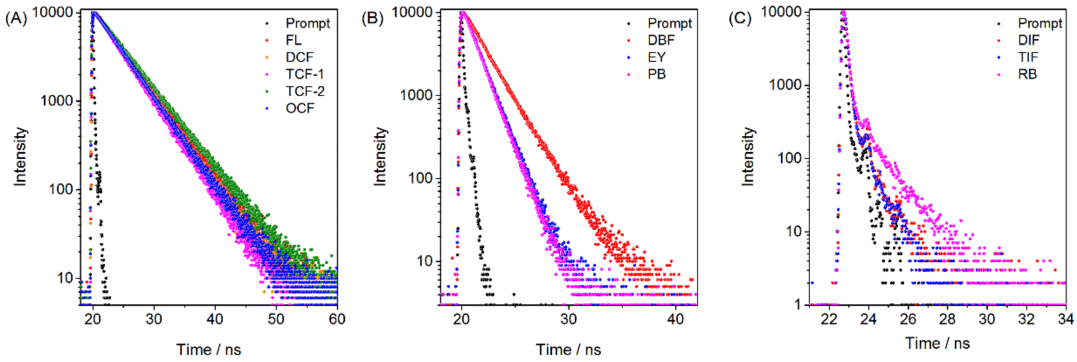

Supplementary Figure 31. The fluorescence lifetime of halogenated- fluorescein derivatives.

S6.3 Relative singlet oxygen quantum yield

The singlet O_2 quantum yield of photosensitizers were measured by Fluolog-3 spectrofluorometer with an integration sphere (IS80, Labsphere) and NIR detector (Hamamatsu H-10330) in this work.

Sample preparation

For better collection of the characterized singlet oxygen phosphorescence emission (1275 nm), CH_3CN and D_2O mixed solvent was used (H_2O can severely quench the singlet oxygen luminescence). The 10 mM stock solutions in CH_3CN and D_2O mixed solvent (V_{CH3CN}:V_{D2O} = 15:1) were first prepared. The stock solutions were diluted to 10 μM, and the final volume was 2 mL. In addition, the CH_3CN and D_2O mixed solvent as blank was also prepared.

Sample measurement

1. Turn on the instrument and the start software, the light source is xenon lamp.
2. From the experiment menu, selected spectra, emission. The $^1O_2$ emission phosphorescence spectra are first measured.
3. The parameters setting are listed:

a. Excitation wavelength: 510 nm

b. Excitation slit: 14 nm

c. Emission wavelength: 1200-1350 nm

d. Emission wavelength: 30 nm

e. Integration time: 1 s

f. Accumulations: 3 average scans

Then, the integrate absorption of sample are collected by CCD (Synapse).

4. Install the integration sphere as normal, put the blank sample cell in the integrating sphere.

5. Choose right attenuation piece and place on excitation side to make the signal strength between 4-5 w. Parameters setting are listed:

a. Excitation wavelength: 510 nm

b. Excitation slit: 3 nm

c. Emission slit: 3 nm

d. Integration time: 0.2 s

e. Accumulations: 10 average scans

Data processing

The peak area of $^1O_2$ phosphorescence emission and integration absorption are integrated by origin 2016.

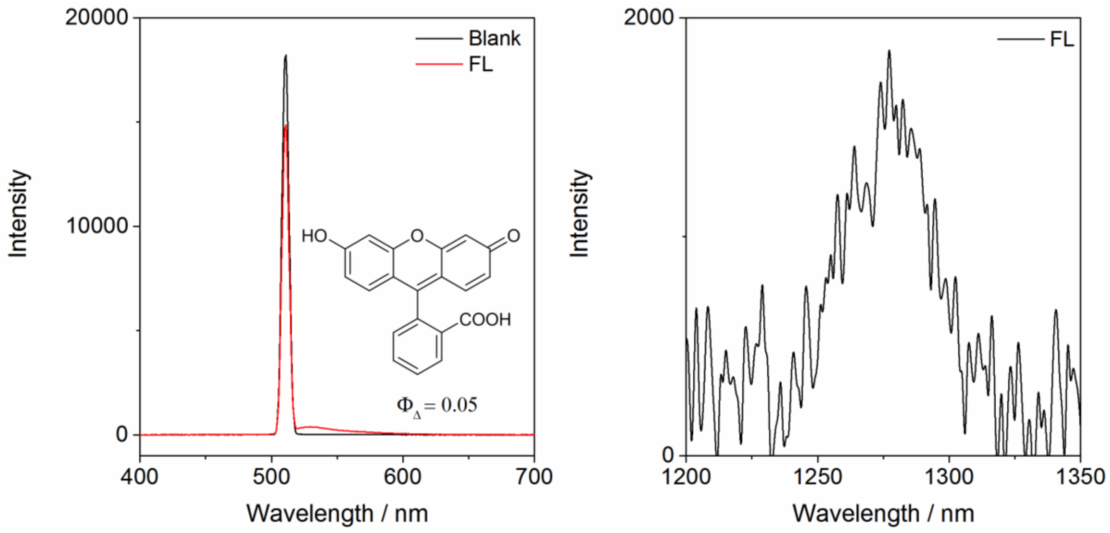

Supplementary Figure 32. The absorbance and the $^1O_2$ emission spectra of FL in mixed solvent (V_{D2O}:V_{CH3CN} = 1:15). (λ_{ex} = 491 nm, c = 10 μM, $\Phi_{overall}$ = 0.01).

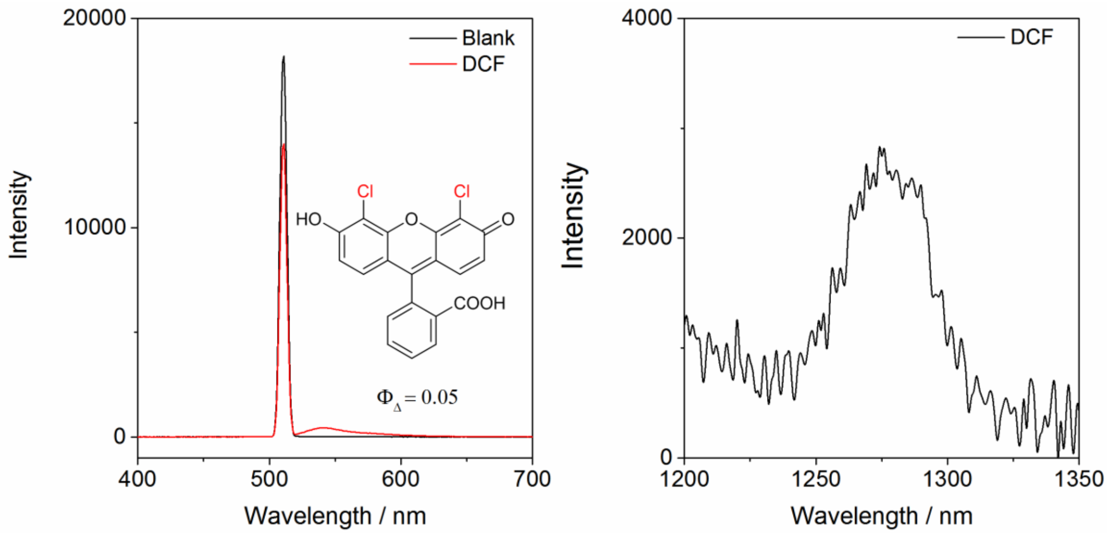

Supplementary Figure 33. The absorbance and the $^{1}O_2$ emission spectra of DCF in mixed solvent (V_{D2O}:V_{CH3CN} = 1:15). (λ_{ex} = 502 nm, c = 10 μM, $\Phi_{overall}$ = 0.04).

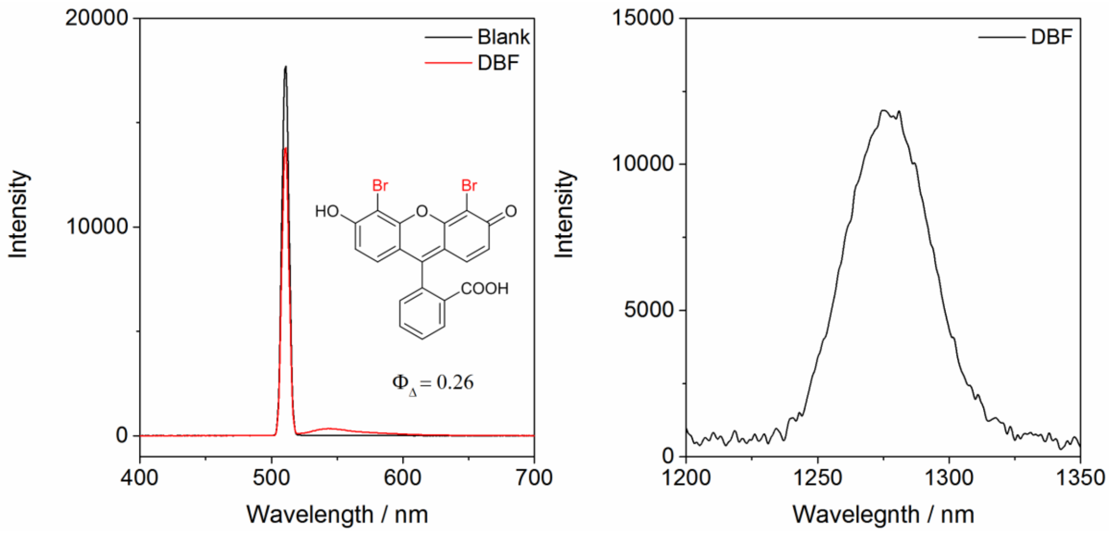

Supplementary Figure 34. The absorbance and the $^{1}O_2$ emission spectra of DBF in mixed solvent (V_{D2O}:V_{CH3CN} = 1:15). (λ_{ex} = 504 nm, c = 10 μM, $\Phi_{overall}$ = 0.19).

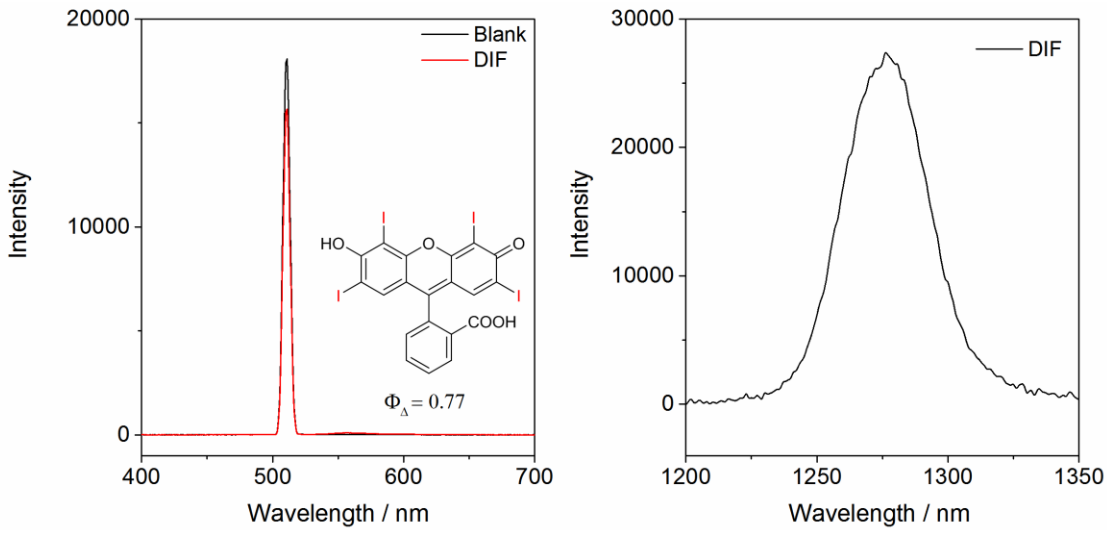

Supplementary Figure 35. The absorbance and the $^1O_2$ emission spectra of DIF in mixed solvent (V_{D2O}:V_{CH3CN} = 1:15). (λ_{ex} = 526 nm, c = 10 μM, $\Phi_{overall}$ = 0.63).

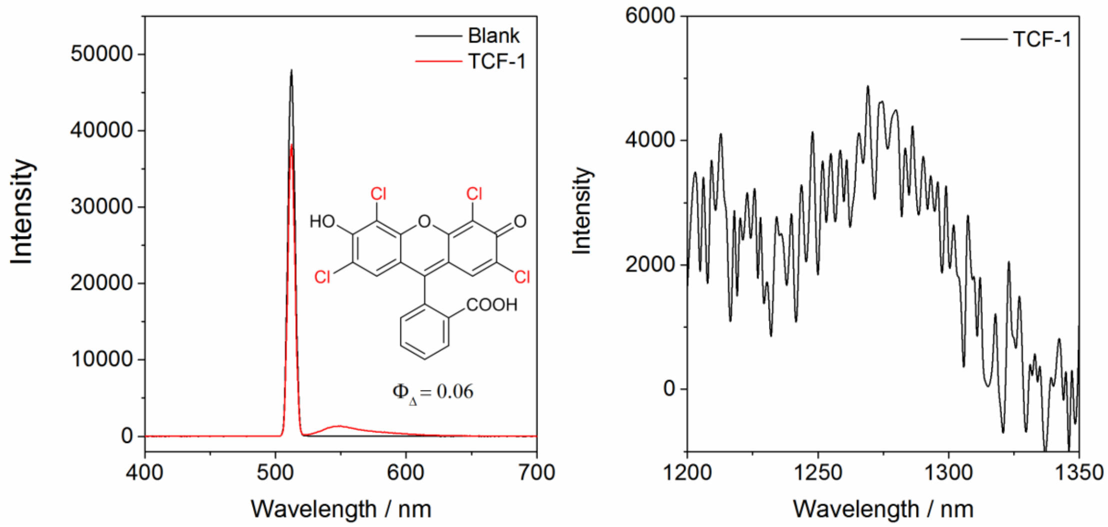

Supplementary Figure 36. The absorbance and the $^1O_2$ emission spectra of TCF-1 in mixed solvent (V_{D2O}:V_{CH3CN} = 1:15). (λ_{ex} = 514 nm, c = 10 μM, $\Phi_{overall}$ = 0.06).

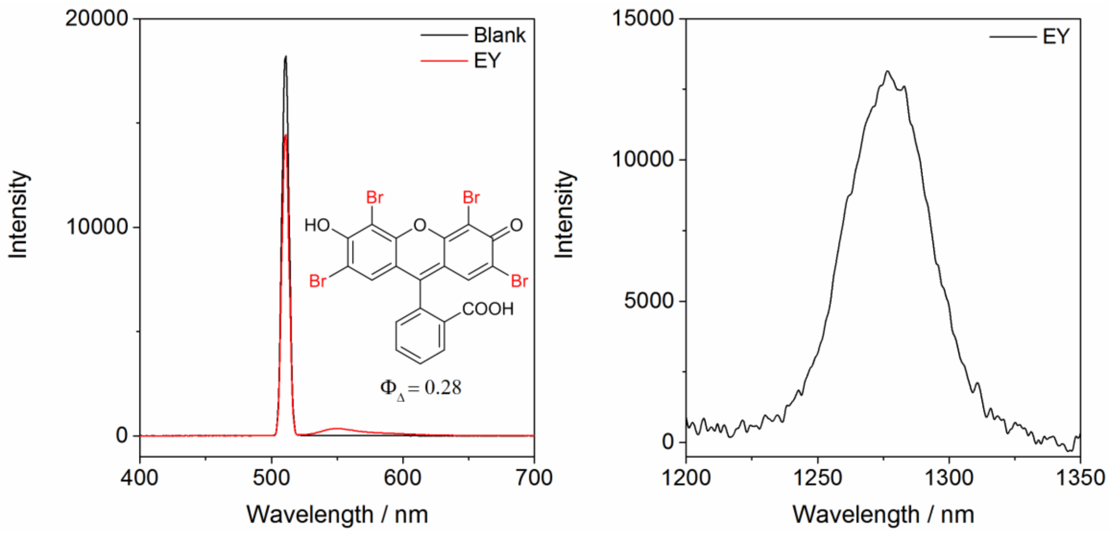

Supplementary Figure 37. The absorbance and the $^1O_2$ emission spectra of EY in mixed solvent (V_{D2O}:V_{CH3CN} = 1:15). (λ_{ex} = 515 nm, c = 10 μM, $\Phi_{overall}$ = 0.20).

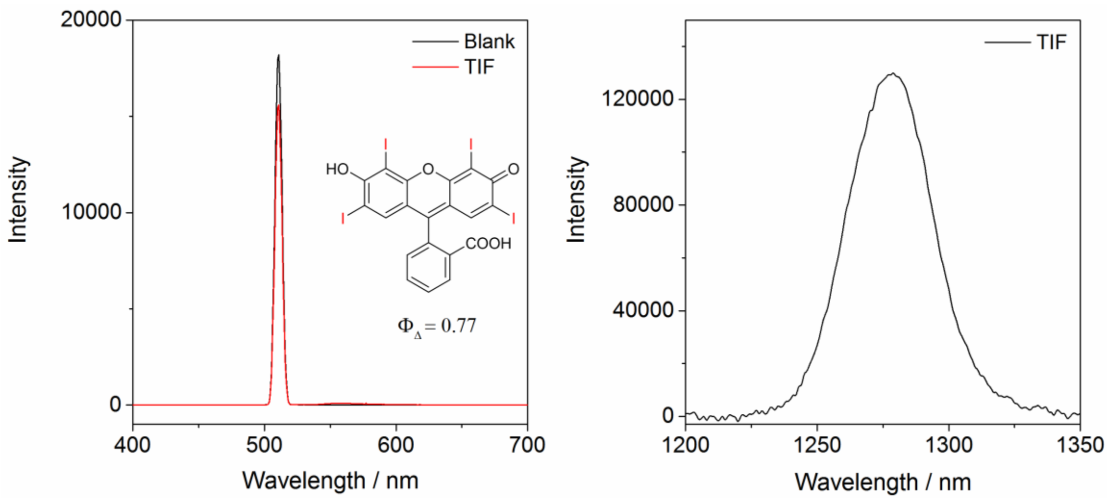

Supplementary Figure 38. The absorbance and the $^1O_2$ emission spectra of TIF in mixed solvent (V_{D2O}:V_{CH3CN} = 1:15). (λ_{ex} = 526 nm, c = 10 μM, $\Phi_{overall}$ = 0.64).

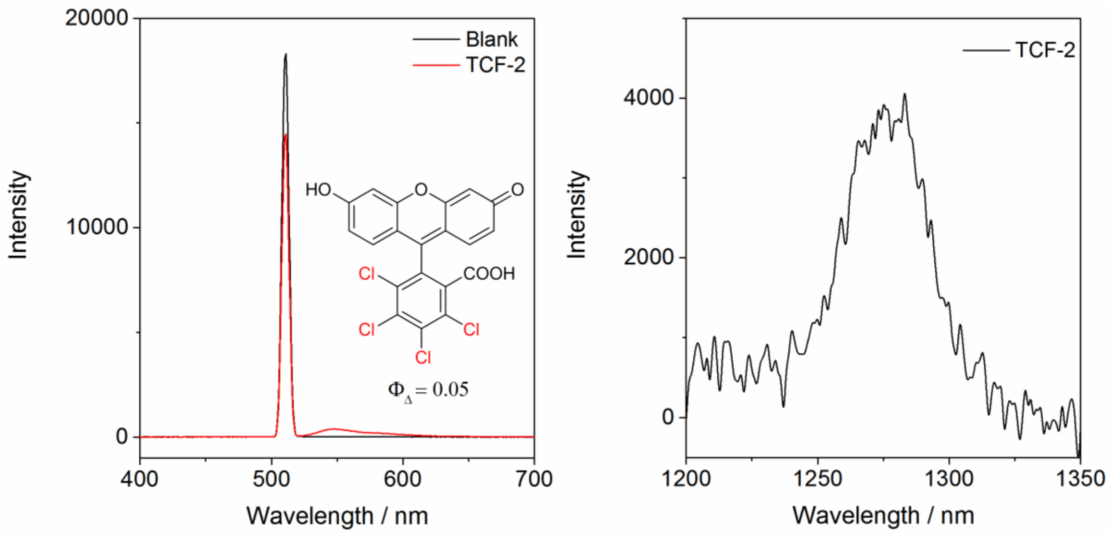

Supplementary Figure 39. The absorbance and the $^1O_2$ emission spectra of TCF-2 in mixed solvent (V_{D2O}:V_{CH3CN} = 1:15). (λ_{ex} = 510 nm, c = 10 μM, $\Phi_{overall}$ = 0.05).

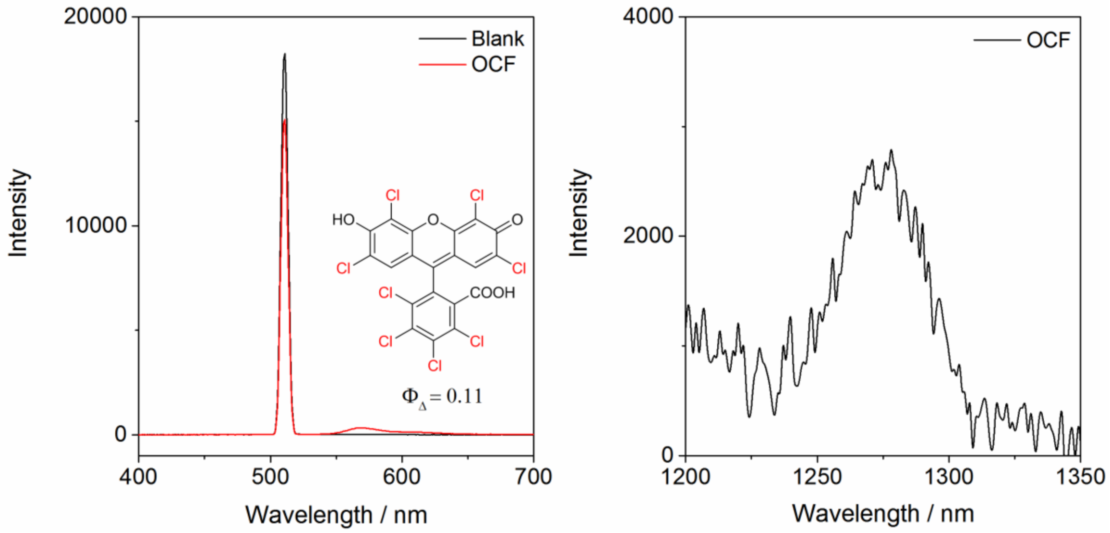

Supplementary Figure 40. The absorbance and the $^1O_2$ emission spectra of OCF in mixed solvent (V_{D2O}:V_{CH3CN} = 1:15). (λ_{ex} = 537 nm, c = 10 μM, $\Phi_{overall}$ = 0.11).

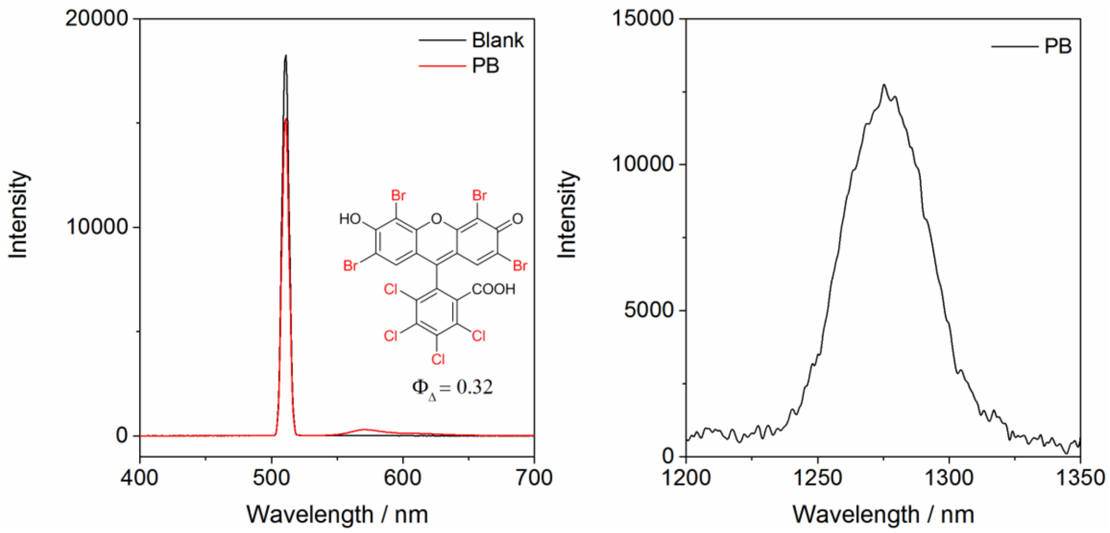

Supplementary Figure 41. The absorbance and the $^{1}O_2$ emission spectra of PB in mixed solvent (V_{D2O}:V_{CH3CN} = 1:15). (λ_{ex} = 539 nm, c = 10 μM, $\Phi_{overall}$ = 0.26).

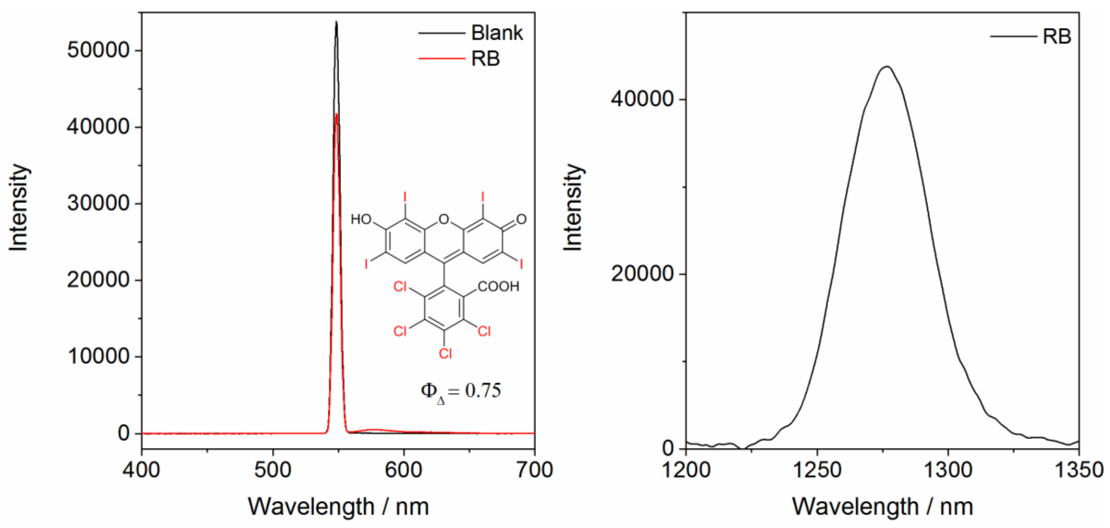

Supplementary Figure 42. The absorbance and the $^{1}O_2$ emission spectra of RB in mixed solvent (V_{D2O}:V_{CH3CN} = 1:15). (λ_{ex} = 548 nm, c = 10 μM, $\Phi_{overall}$ = 0.75).

S6.4 Transient absorption spectra

Transient absorption spectra of DIF TIF and RB were measured in N_2-saturated deionized water upon 532 nm laser excitation. The sample concentration was 10 μM.

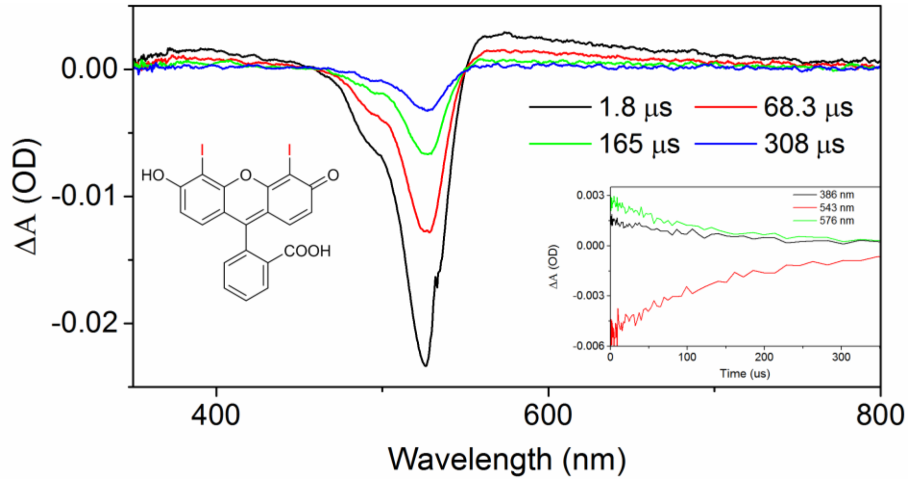

Supplementary Figure 43. Transient absorption spectra of DIF upon 532 nm laser excitation. The inset shows the time profile for ΔOD recorded at 386 nm, 543nm and 576 nm.

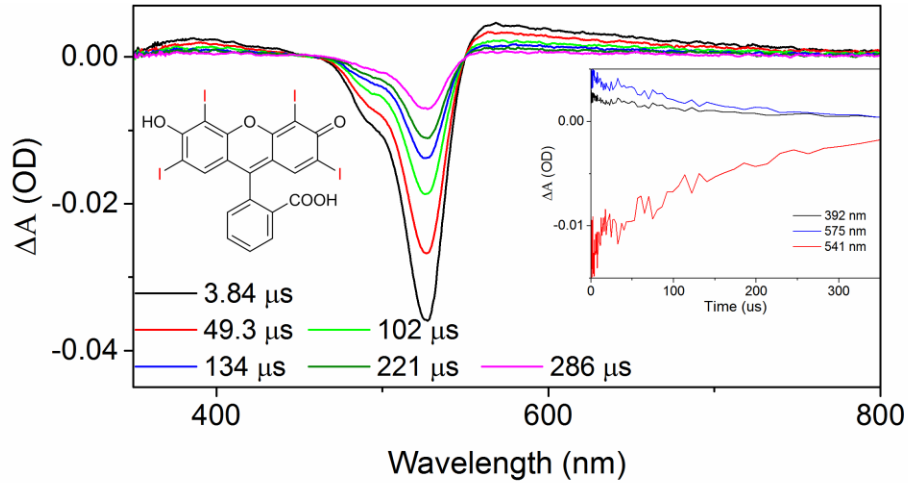

Supplementary Figure 44. Transient absorption spectra of TIF upon 532 nm laser excitation. The inset shows the time profile for ΔOD recorded at 392 nm, 541nm and 575 nm.

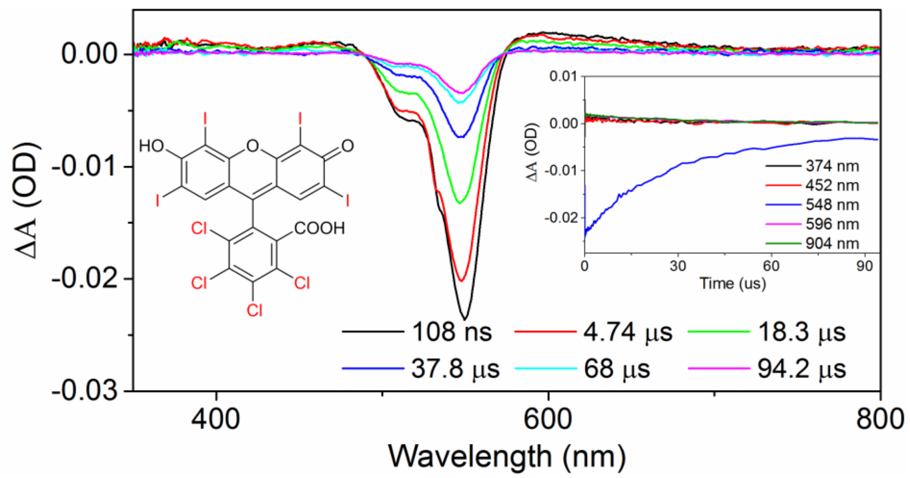

Supplementary Figure 45. Transient absorption spectra of RB upon 532 nm laser excitation. The inset shows the time profile for ΔOD recorded at 374 nm, 452 nm, 548 nm, 596 nm and 904 nm.

The decay rate constants (k_{dec}), radiative rate constants (k_{rad}) and non-radiative rate constants (k_{nr}) were calculated according to the following equations:[5]

$$\emptyset = \frac{k_r}{k_r + k_{nr}} \quad (1)$$

$$\tau = \frac{1}{k_r + k_{nr}} \quad (2)$$

$$k_{nr} = \frac{1-\emptyset}{\tau} \quad (3)$$

S6.5 Photophysical properties of the halogenated-fluorescein derivatives

Supplementary Table 4. Photophysical Properties of Halogenated-fluorescein derivatives.

Compd	λ_{Abs} (nm)[a]	λ_{em} (nm)[a]	λ_{Ph} (nm)[a]	$pH_{0.5}$[b]	Singlet oxygen yield ($\Phi_{\triangle}$)[c]	Φ_{FL}[d]	τ_{fl} (ns)[e]	$k_{dec}\times10^8$ (s^{-1})	$k_{rad}\times10^8$ (s^{-1})	$k_{nr}\times10^8$ (s^{-1})
FL	491	515	638	6.09	0.05	0.858	4.58	2.18	1.87	0.31
DCF	502	529	645	4.77	0.05	0.668	4.33	2.31	1.54	0.77
DBF	504	531	685	4.63	0.26	0.303	1.99	5.03	1.52	3.50
DIF	527	549	699	4.13	0.77	0.001	0.01	1000.00	1.00	999.00
TCF-1	515	536	655	2.79	0.06	0.680	4.09	2.44	1.66	0.78
EY	515	556	687	3.07	0.28	0.197	1.30	7.69	1.52	6.17
TIF	527	551	701	4.07	0.77	0.003	0.01	1000.00	3.00	997.00
TCF-2	511	531	637	5.89	0.05	0.854	4.87	2.05	1.75	0.30
OCF	537	556	676	3.41	0.11	0.597	4.33	2.31	1.38	0.93
PB	539	560	730	3.45	0.32	0.170	1.25	8.00	1.36	6.64
RB	550	570	732	3.99	0.75	0.003	0.01	1000.00	3.00	997.00

[a] Measured in 2% DMSO; [b] Measured in 2% DMSO citric acid-disodium hydrogen phosphate buffer; [c] Measured in a mixed solvent of D_2O and CH_3CN (V_{D2O}: V_{CH3CN} = 1: 15); [d] The solution of samples were prepared to be 5 μM in 0.5% (v/v) DMSO, and diluted with 0.1 M NaOH.

Section S7. Basic Performances of TIF in Photodynamic Bacteria Inactivation

S7.1 Bacteria inactivation performance of different photosensitizers

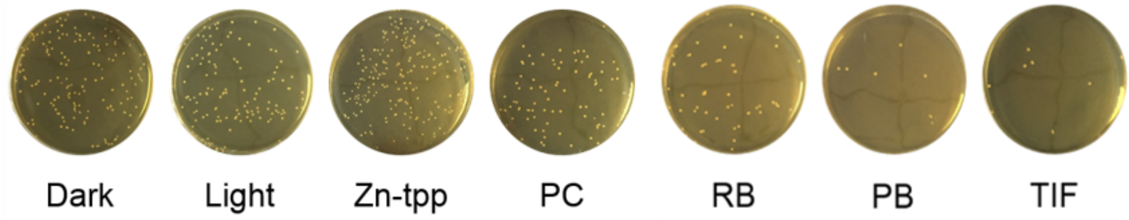

Supplementary Figure 46. Growth inhibition of *L. plantarum* by 0.5 μM different photosensitizers in 10 min (pH = 2.5).

S7.2 International standards and limits of TIF

Supplementary Table 5. The information about the food addictive TIF in the national standards of various agencies.

Agencies	Name	Maximum use level	Standards
US FDA	FDC Red No 3	GMP	CFR-Title 21, Part 74
European Union	Erythrosine BS	150 mg/kg	European Parliament and Council Directive 94/36/EC|ANNEX III
Chinese National Standard	Erythrosine B	50 mg/kg	GB 2760-2011

GMP: Good Manufacturing Practice

S7.3 *Lactobacillus* inactivation performance of TIF at different pH

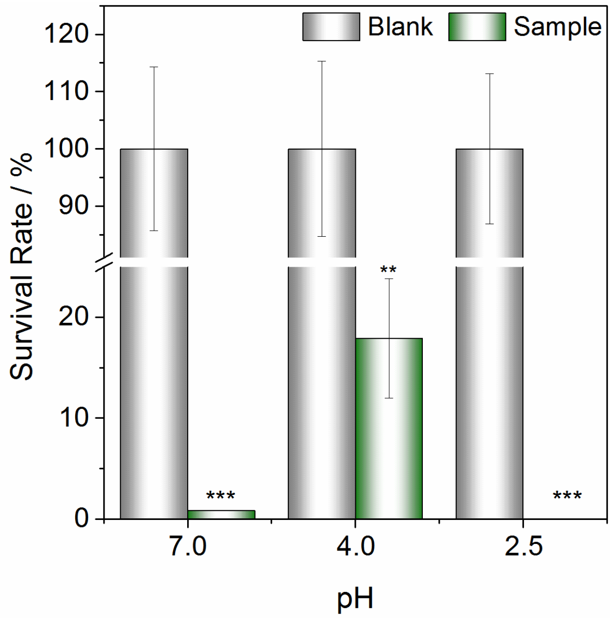

Supplementary Figure 47. Growth inhibition of *L. plantarum* for 0.5 μM different pH in 10 min (*P < 0.05, **P < 0.01, ***P < 0.001). Error bars = Standard Deviation (n=3).

S7.4 Identification of specific ROS generated from TIF

For the collection of the EPR signal of •OH and $O_2^{\bullet-}$, 5,5-dimethyl-1-pyrroline-N-oxide (DMPO) was employed as the trapping agent in H_2O and MeOH, respectively. For ROS scavenging, β-carotene, mannite, p-benzoquinone can eliminate the generated $^1O_2$, •OH and $O_2^{\bullet-}$, respectively. *L. plantarum* were shaken overnight at 37 ℃, 170 r/min. After centrifugation and washing with sterile water, the activated *L. plantarum* were diluted for 100 times with PBS buffer. Photosensitizer and scavengers were added to the bacteria solution, and then illuminated for 2 min. The solution (10 μL) was added to the medium and cultured at 37 ℃ for 24h.

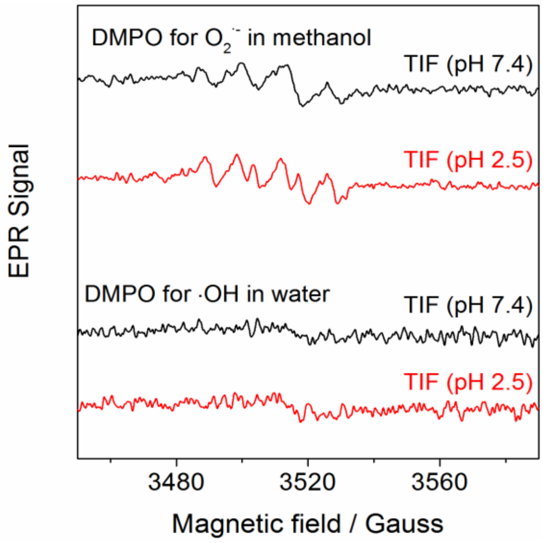

Supplementary Figure 48. EPR spectra of TIF (LED light time, 60 s) in the presence of DMPO (a specific spin trap for •OH and $O_2^{\bullet-}$).

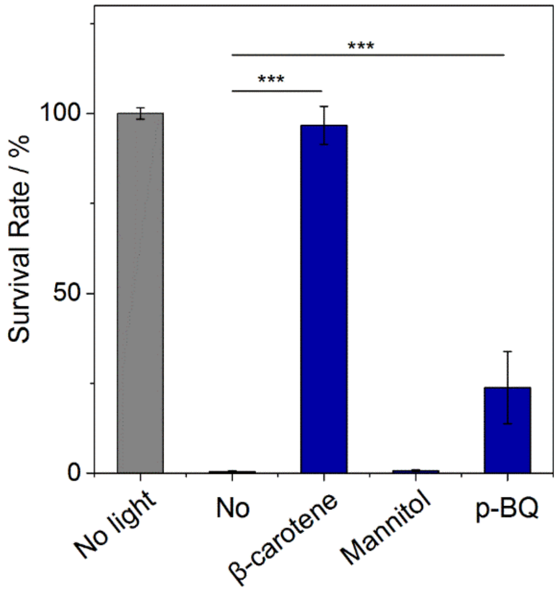

Supplementary Figure 49. Identification of specific ROS generated from photosensitization of TIF with scavengers (*$P < 0.05$, **$P < 0.01$, ***$P < 0.001$). Error bars = Standard Deviation (n=3).

S7.5 The surface charge of E. coli in different solvents

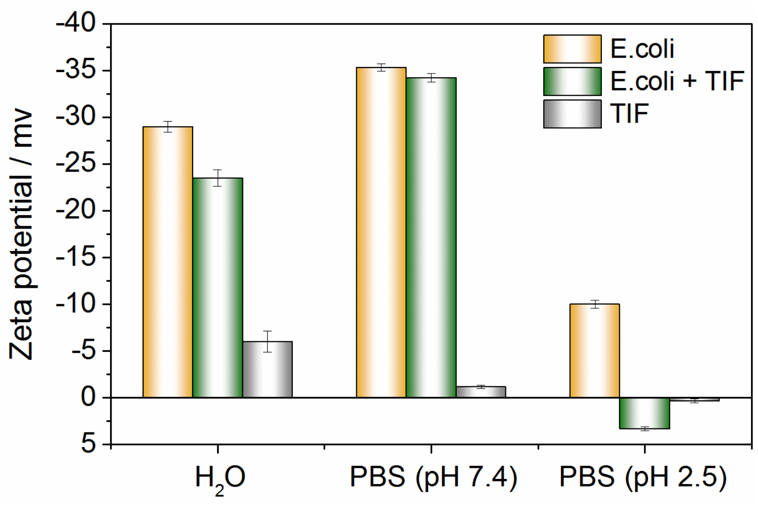

Supplementary Figure 50. Zeta potentials of *E.coli*, *E.coli* + TIF and TIF in different solvent. Error bars = Standard Deviation (n=3).

Section S8. Inactivation performance of TIF for different bacterial strains

Supplementary Table 6. The essential information of microorganisms in these work.

	Microorganism name	pH	Culture medium	Incubation time (h)	Culture temperature (ºC)
Gram-positive (G+)	*Lactobacillus plantarum*	2.5	MRS	24	37
	Alicyclobacillus acidoterrestris	2.5	-	24	50
	Staphylococcus aureu	2.5	LB	12	37
	Methicillin-resistant Staphylococcus aureu	2.5	LB	12	37
Gram-negative (G-)	*Escherichia coli*	2.5	LB	12	37
	Salmonella enterica	2.5	LB	12	37
	Helicobacter pylori	2.5	Columbia, Brain heart immersion, sheep blood	84	37
Fungus	*Candida albicans*	3.0	PDA	12	28

MRS: Deman Rogosa Sharpe; LB: Lysogeny broth; PDA: Potato Dextrose Water.

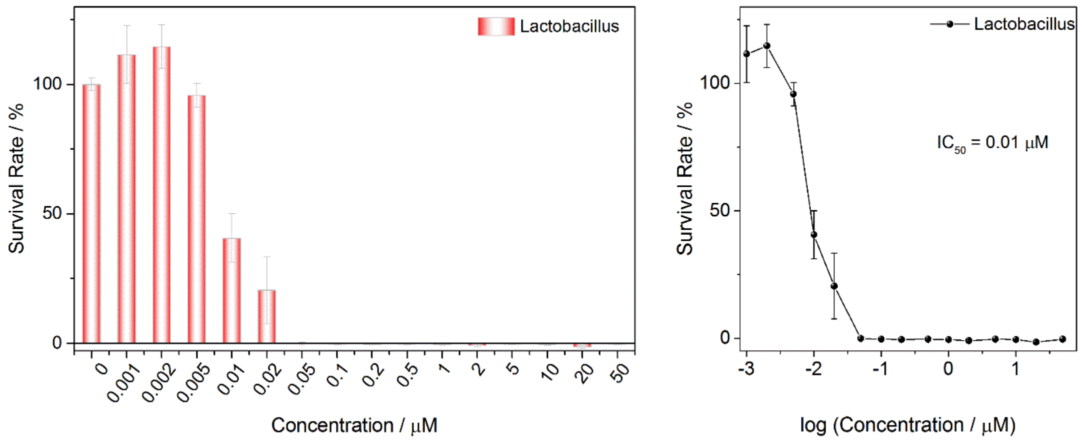

Supplementary Figure 51. Growth inhibition of *L. plantarum* by different concentrations of TIF. Irradiation: 520 nm green LED (6 mW/cm^2), 10 min. Error bars = Standard Deviation (n=6).

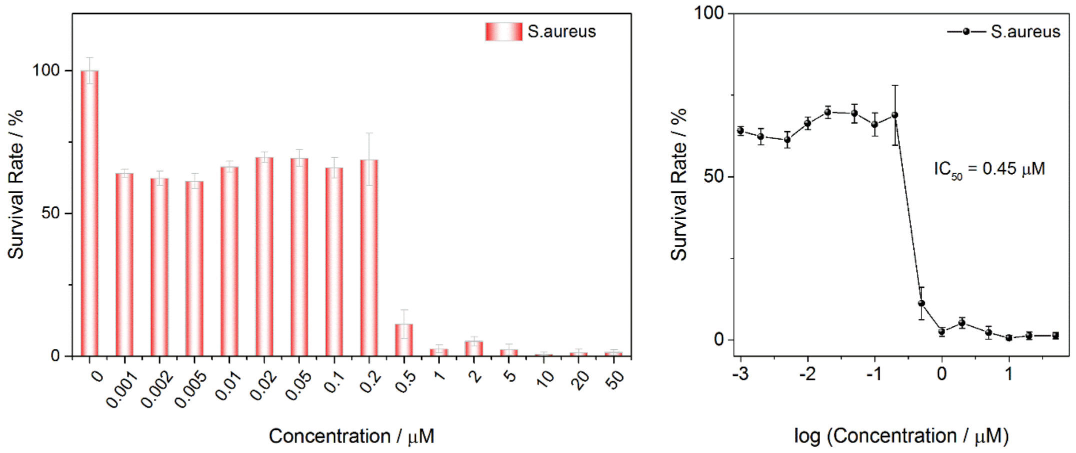

Supplementary Figure 52. Growth inhibition of *S. aureus* by different concentrations of TIF. Irradiation: 520 nm green LED (6 mW/cm^2), 10 min. Error bars = Standard Deviation (n=6).

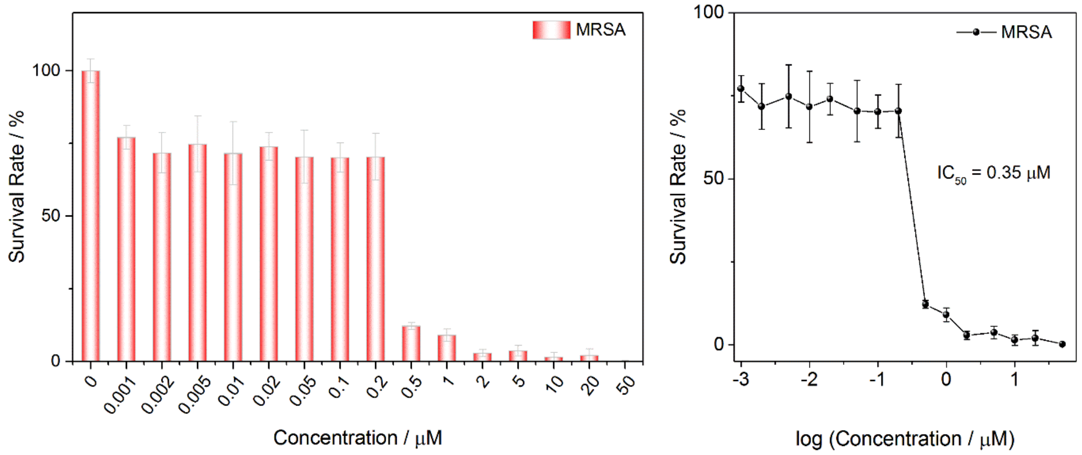

Supplementary Figure 53. Growth inhibition of *MRSA* by different concentrations of TIF. Irradiation: 520 nm green LED (6 mW/cm^2), 10 min. Error bars = Standard Deviation (n=6).

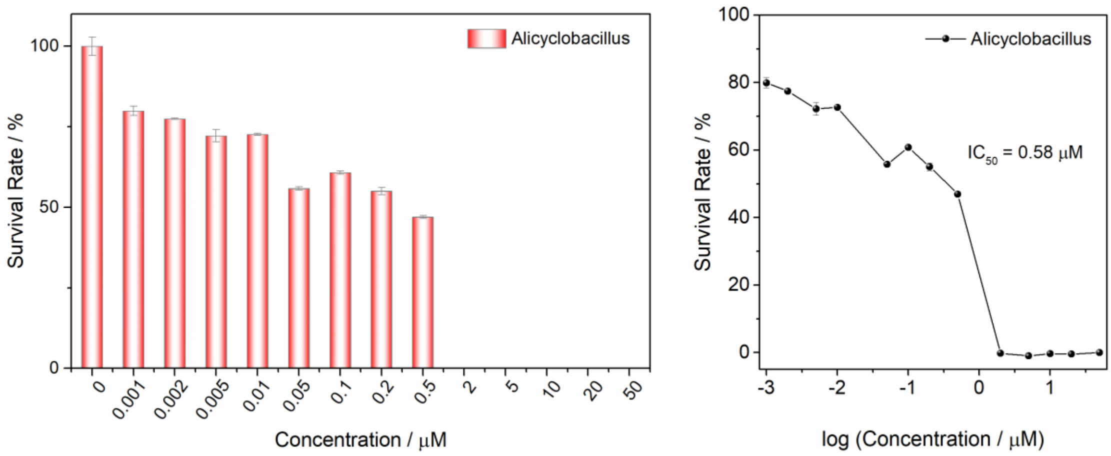

Supplementary Figure 54. Growth inhibition of *A. acidoterrestris* by different concentrations of TIF. Irradiation: 520 nm green LED (6 mW/cm^2), 10 min. Error bars = Standard Deviation (n=6).

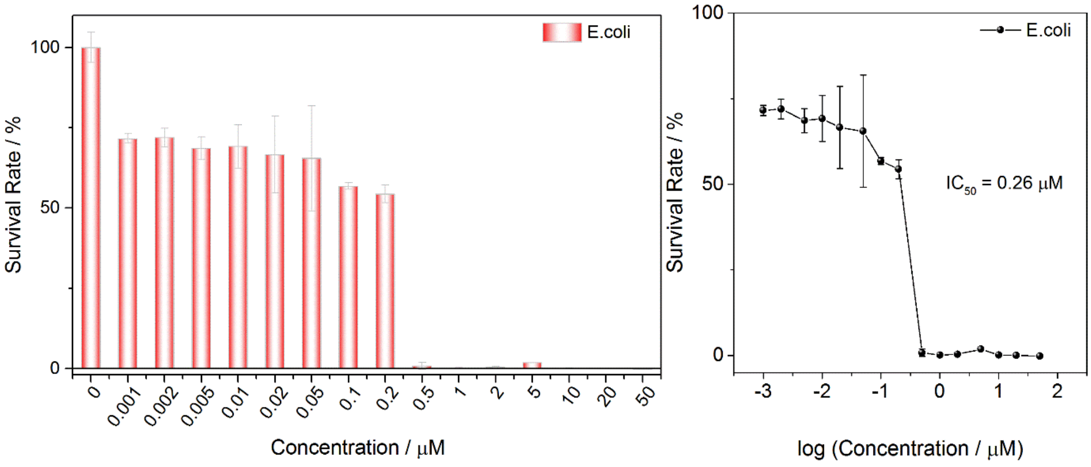

Supplementary Figure 55. Growth inhibition of *E. coli* by different concentrations of TIF. Irradiation: 520 nm green LED (6 mW/cm^2), 10 min. Error bars = Standard Deviation (n=6).

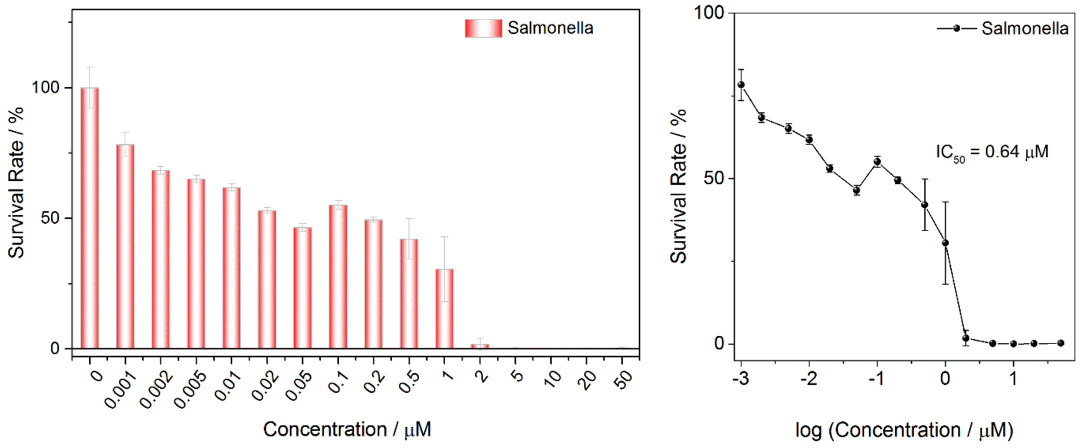

Supplementary Figure 56. Growth inhibition of *Salmonella* by different concentrations of TIF. Irradiation: 520 nm green LED (6 mW/cm^2), 10 min.

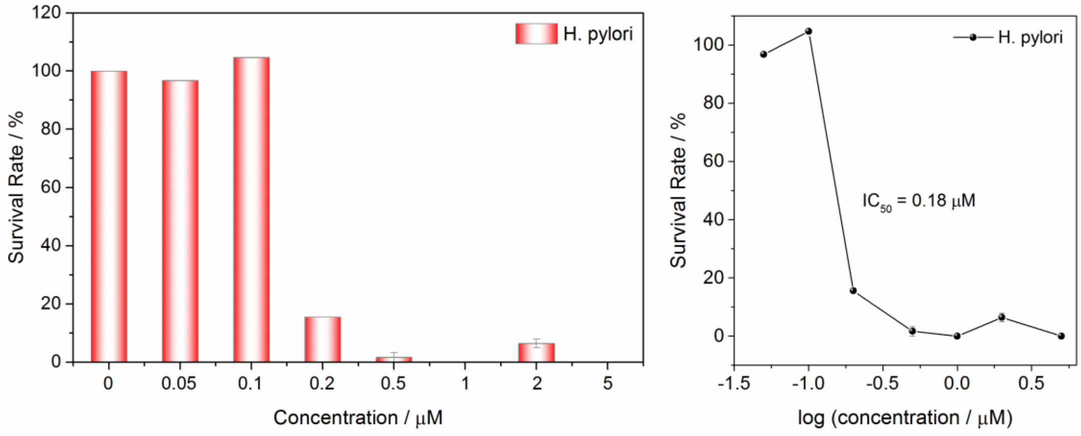

Supplementary Figure 57. Growth inhibition of *H. Pylori* by different concentrations of TIF. Irradiation: 520 nm green LED (6 mW/cm^2), 10 min. Error bars = Standard Deviation (n=3).

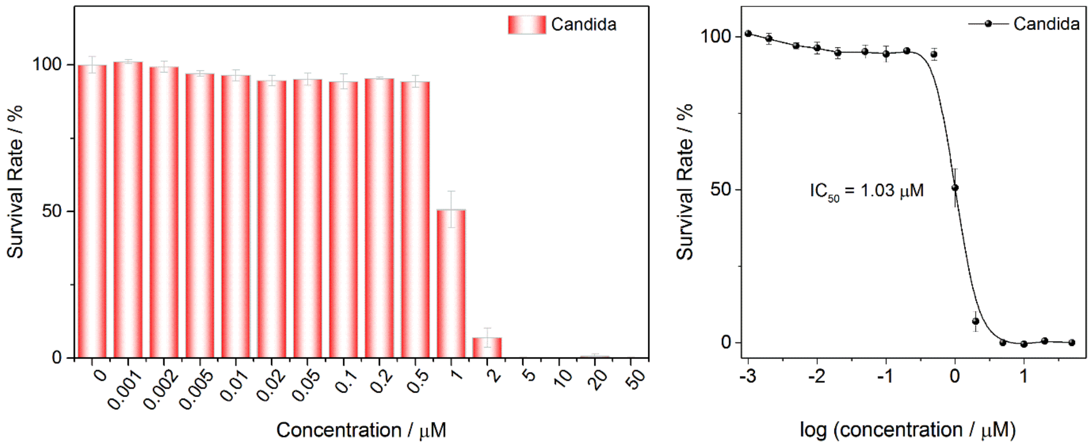

Supplementary Figure 58. Growth inhibition of *C. albicans* by different concentrations of TIF. Irradiation: 520 nm green LED (6 mW/cm^2), 10 min. Error bars = Standard Deviation (n=6).

Supplementary Table 7. Comparison of the photodynamic bacteria inactivation performances of different photosensitizers.

	PSs	Organism	Dose	Light Dose	Irradiation time	Inhibition ratio	Ref
Organic molecules	Brominated-BF_2	*MRSA*	4.3 μM	16 J/cm^2	10 min	> 99.9%	[6]
	BODIPY	*E.coli*	5.0 μM	1.3 J/cm^2	5 min	3.6 (log_{10})	[7]
	Methylene Blue	*MRSA*	100 mg/L	360 J/cm^2	-	4.7 (log_{10})	[8]
Coordination compounds	$[Ru(bpy)_2(dppn)]^{2+}$	*E.coli*	0.5 μM	-	20 min	5.4 (log_{10})	[9]
	BLRu	*MRSA*	1 μM	84 J/cm^2	-	> 80%	[10]
Nano-materials	MoS_2	*E.coli*	1.6 mg/L	Real Sunlight	20 min	> 99.999%	[11]
	C-dots	*E.coli*	5.0 μM	3 V/3 W	60 min	> 92%	[12]
	ZIF-8	*E.coli*	0.15 mg/cm^2	-	30 min	> 99.99%	[13]
This work	TIF	*E.coli*	0.5 μM	3 V/3 W, 6 mW/ cm^2	10 min	> 99.3%	
	TIF	*MRSA*	2.0 μM	3 V/3 W, 6 mW/ cm^2	10 min	> 97.1%	

Section S9. Photodynamic Antibacterial Inactivation Performance of TIF in Acidic Juices

S9.1 Biocompatibility of TIF

L929 mouse fibroblasts cell line was chosen to assess the biocompatibility of TIF with a standard Cell Counting Kit-8 (CCK-8) assay. Different concentrations of TIF were incubated with L929 cells for 24 h.

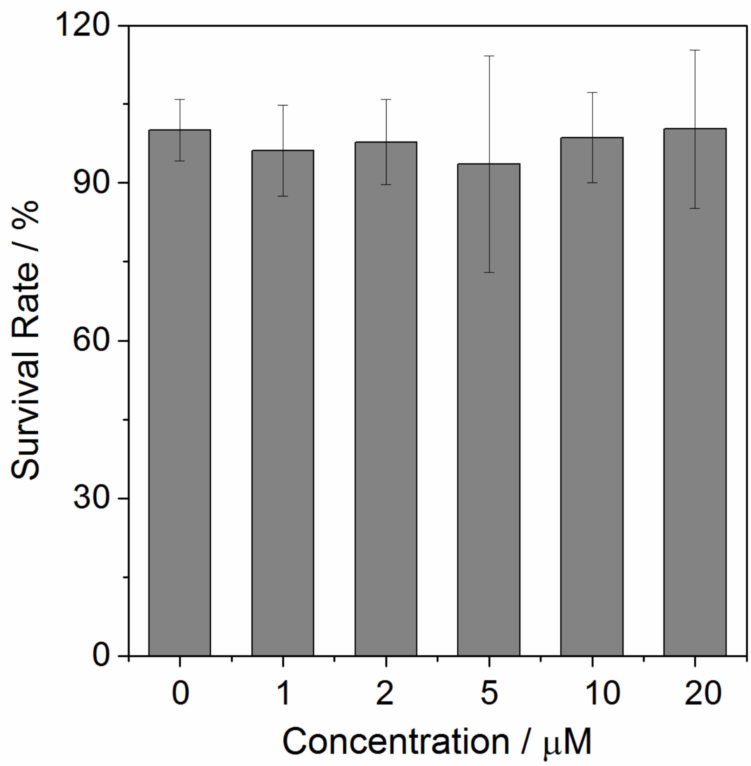

Supplementary Figure 59. The biocompatibility of TIF evaluated with CCK-8 assay. Error bars = Standard Deviation (n=6).

S9.2 The antimicrobial activity of TIF under different LED irradiation

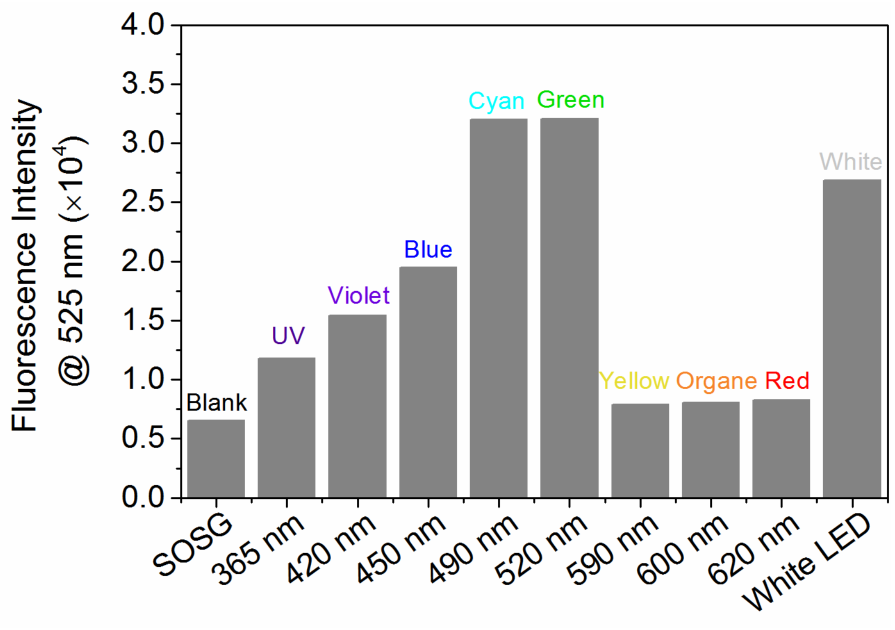

Supplementary Figure 60. Irradiation Wavelength-dependent generation of $^1O_2$ from TIF evaluated by the SOSG fluorescent probe. Irradiation time: 2 min.

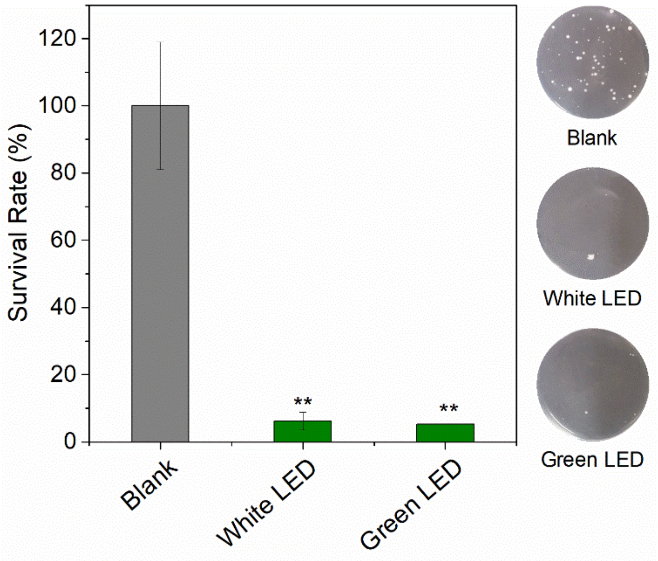

Supplementary Figure 61. Survival rates of microbial in passion fruit lemon juice after treated with different light sources (520 nm and white LED 3 V/3 W, 23 mW/cm^2) and TIF. Error bars = Standard Deviation (n=3).

S9.3 Photodynamic antibacterial in juices

Supplementary Table 8. Common acidic beverages and their pH values.

	categories	pH
Coca Cola	Carbonated beverage	2.45
Powerade	Function beverage	2.77
Orange juice	Fruit and vegetable Juice beverage	3.12
Yogurt	Dairy beverage	4.24

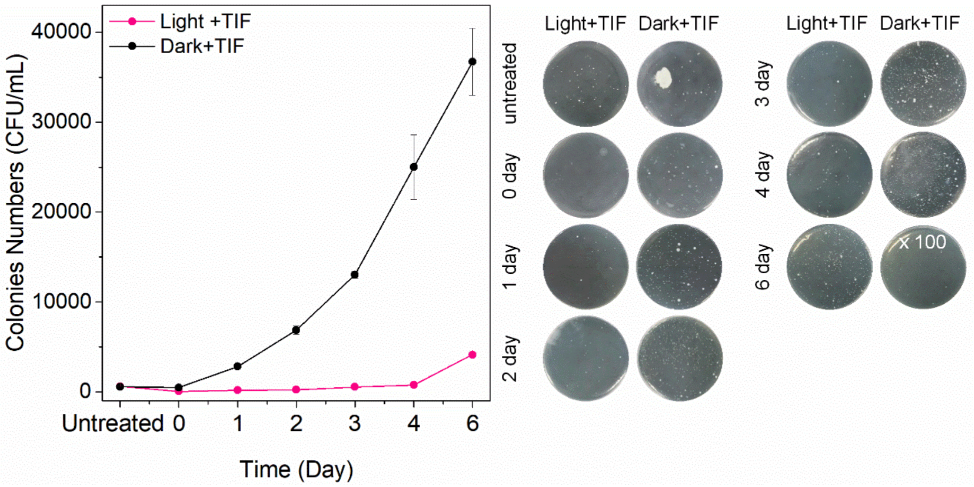

Supplementary Figure 62. Growth inhibition of bacteria in passionfruit lemon juice (pH 2.8) by 10 μM TIF. Irradiation: Xenon lamp (100 mW/cm^2), 20 min. Error bars = Standard Deviation (n=3).

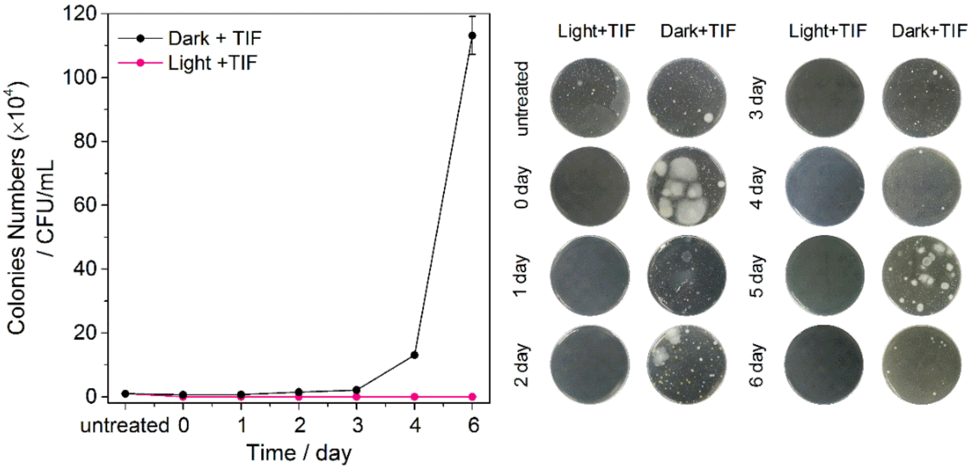

Supplementary Figure 63. Growth inhibition of bacteria in grape juice (pH 4.2) by 10 μM

TIF. Irradiation: Xenon lamp (100 mW/cm^2), 20 min. Error bars = Standard Deviation (n=3).

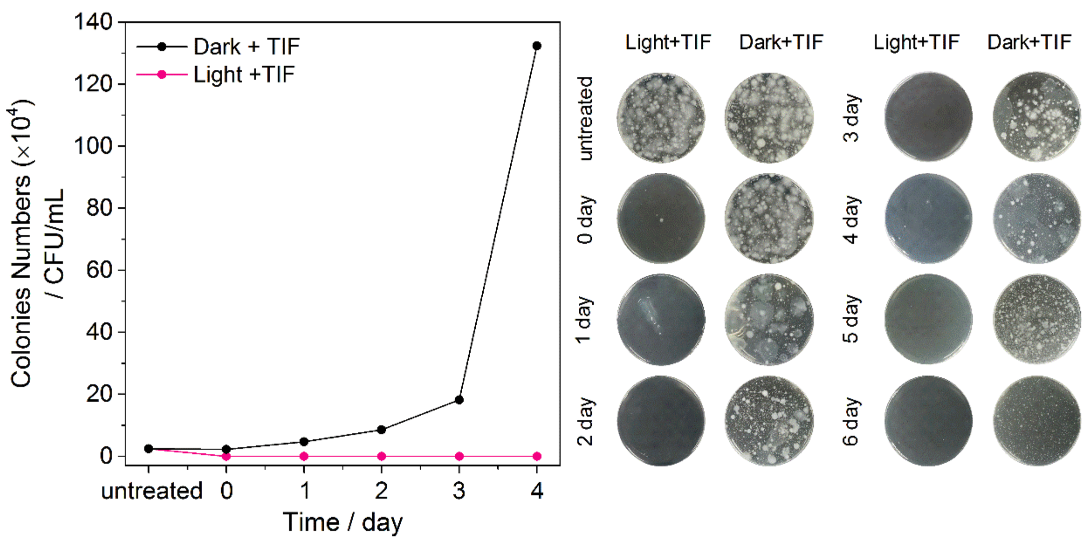

Supplementary Figure 64. Growth inhibition of bacteria in tomato juice (pH 4.6) by 10 μM TIF. Irradiation: Xenon lamp (100 mW/cm^2), 20 min. Error bars = Standard Deviation (n=3).

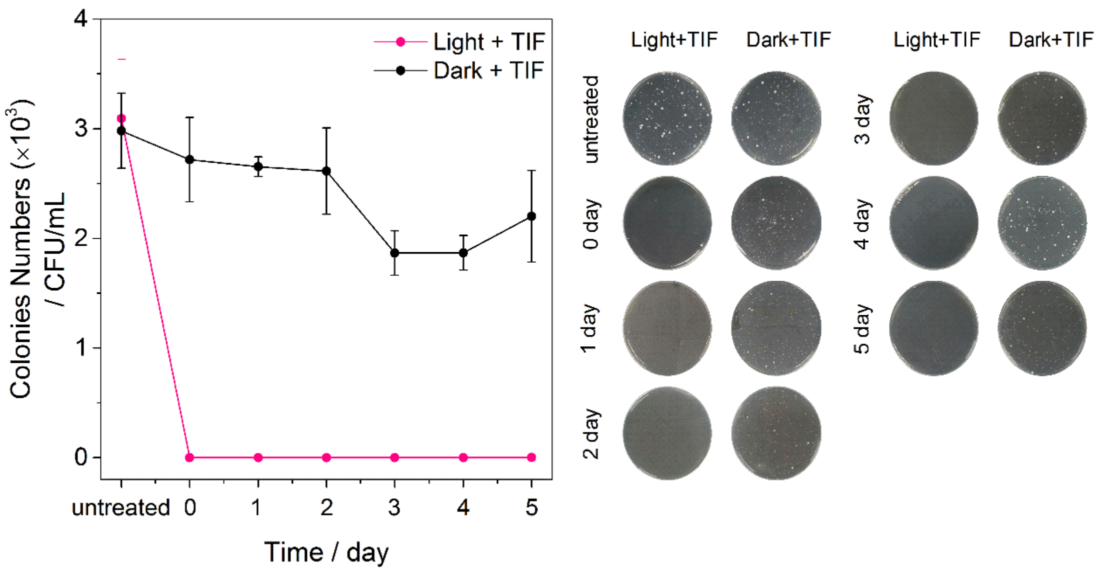

Supplementary Figure 65. Growth inhibition of bacteria in commercial grape juice (pH 3.6) by 10 μM TIF. Irradiation: Xenon lamp (100 mW/cm^2), 10 min. Error bars = Standard Deviation (n=3).

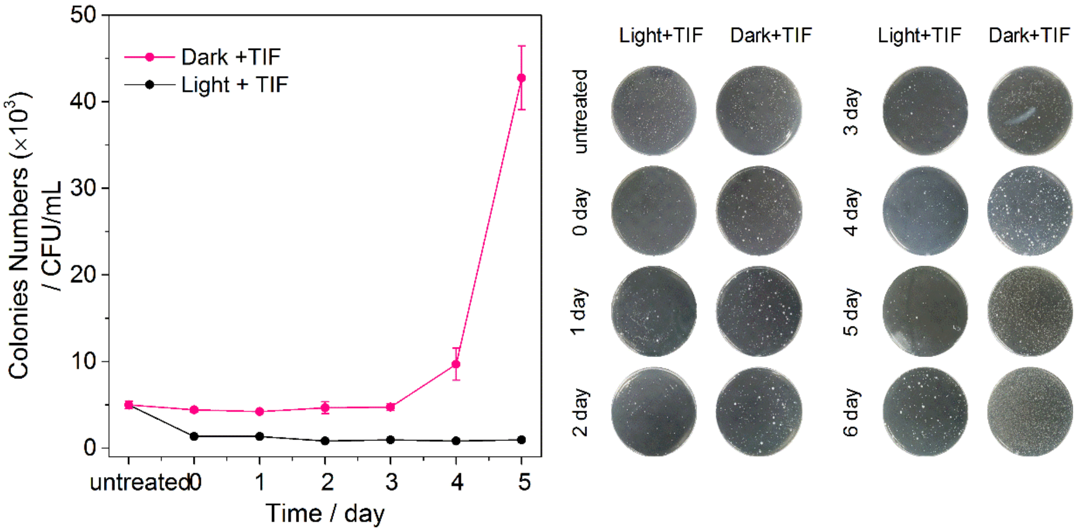

Supplementary Figure 66. Growth inhibition of commercial grapefruit juice (pH 3.6) by 10 μM TIF. Irradiation: Xenon lamp (100 mW/cm^2), 10 min. Error bars = Standard Deviation (n=3).

The freshly squeezed passionfruit lemon juice, strawberry juice and tomato juice treated with photodynamic sterilization were first prepared and stored at 4 °C. The colonies number were measured followed by GB 4789.2-2016.

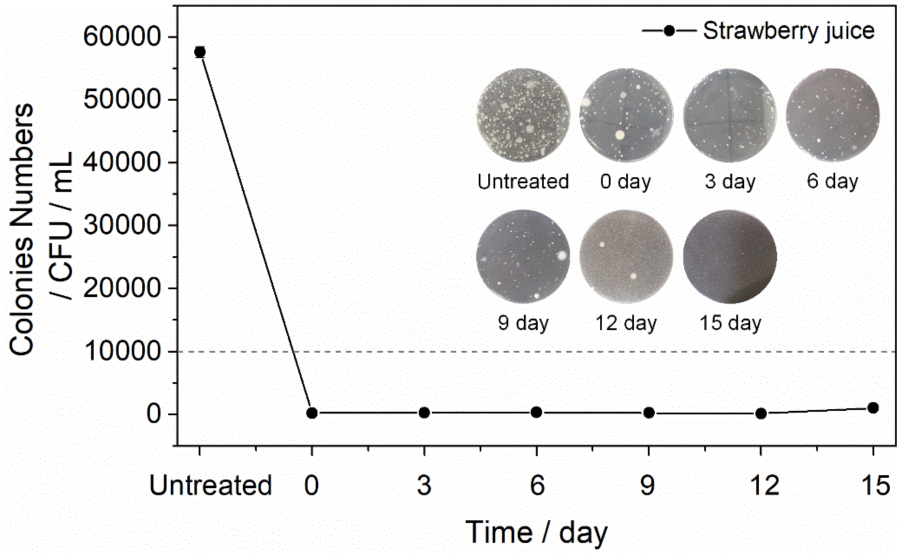

Supplementary Figure 67. Bacterial colony number in strawberry juice (pH 3.5) treated with TIF-based photodynamic sterilization at different time intervals. Error bars = Standard Deviation (n=3).

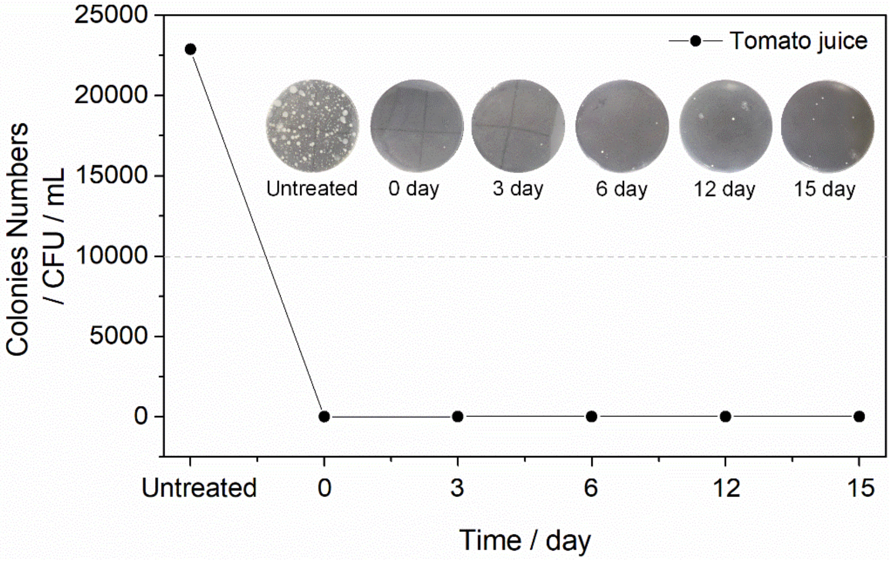

Supplementary Figure 68. Bacterial colony number in tomato juice (pH 4.6) treated with TIF-based photodynamic sterilization at different time intervals. Error bars = Standard Deviation (n=3).

S9.4 Long-term colonies numbers monitoring

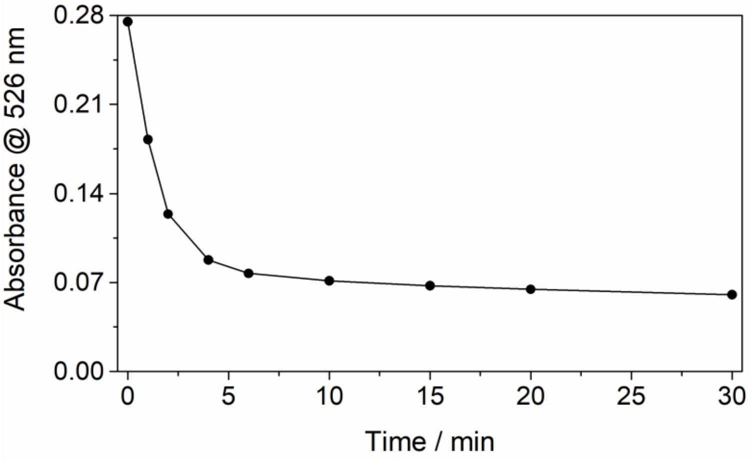

Supplementary Figure 69. Photo-bleaching test of TIF in pH 3.0 PBS (green LED, 50 mW/cm^2). Error bars = Standard Deviation (n=3).

S9.5 Antioxidants contents in juices

Ascorbic acid content

The content of ascorbic acid was measured according to the method recommended by

the Chines National Standard (GB 5009.86-2016). The detailed procedures were as follows:

(1) The treated juice was mixed with the same weight of the metaphosphoric acid-acetic acid solution and stirred evenly.

(2) The pH of the mixture (1, 20 g) was adjusted to pH 1.2 with metaphosphoric acid-acetic acid solution, and then diluted to volume (100 mL).

(3) 2 g activated carbon was added to the above mixture (2, 50 mL), followed by shaking for 1 min and filtration.

(4) The mixed solution (3, 10 mL) was added to the sodium acetate solution (5 mL, 500 g/mL). After incubation for 15 min, the mixture was diluted to volume (100 mL).

(5) The sample (4, 2 mL) was added to the *o*-phenylenediamine solution (5 mL, 200 mg/mL) in dark, and the absorbance at 420 nm was measured immediately.

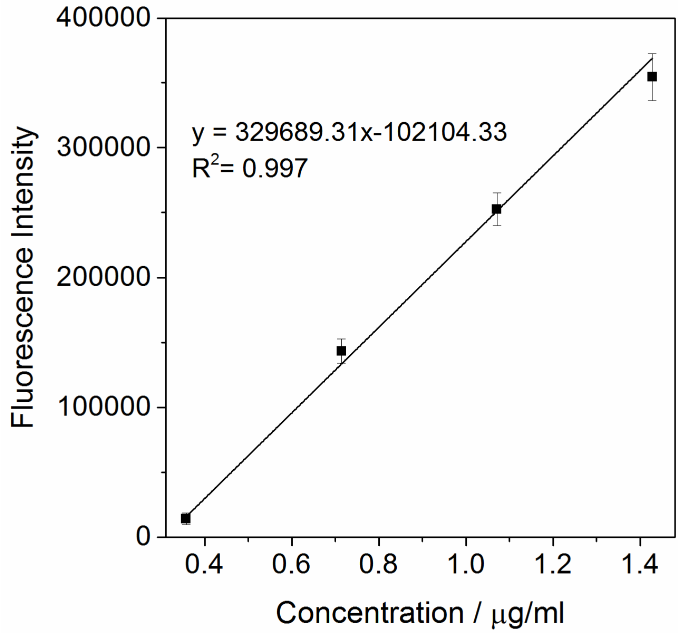

Supplementary Figure 70. The standard curve of ascorbic acid. Error bars = Standard Deviation (n=3).

Total phenolic content

Total phenolic content (TPC) was measured with the Folin-Ciocalteu method. The detailed procedures were as follows:

(1) The treated juice (200 μL) was added to 80 % methanol (800 μL, containing 1%

HCl) and centrifuged at 8000 r/min for 10 min.

(2) 300 μL of the supernatant was add to 750 μL FC reagent (1:5 v/v) and incubated for 5 min.

(3) 750 μL of 7.5% Na_2CO_3 solution was added to the mixed solution (2).

(4) After incubation for 90 min, the absorbance at 760 nm were measured. Calibration was constructed with gallic acid as the standard.

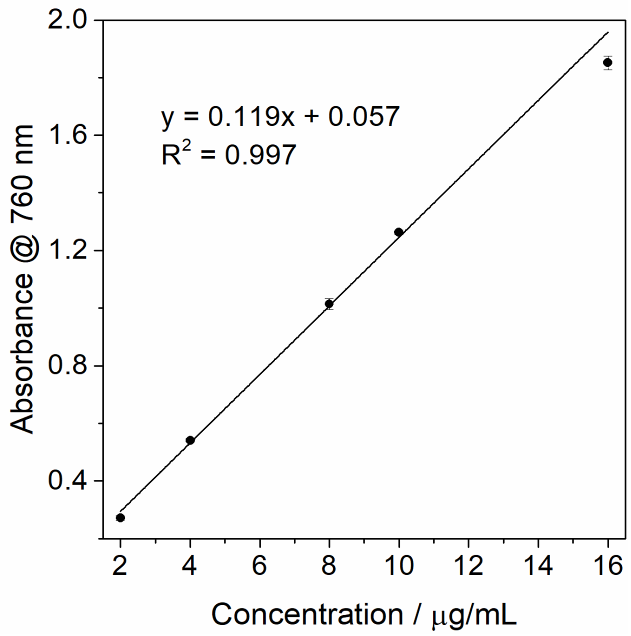

Supplementary Figure 71. The standard curve of gallic acid. Error bars = Standard Deviation (n=3).

Total flavonoid content

Total flavonoid content (TFC) was measured based the method developed by Ghafar et al.[14] Briefly, 2% $AlCl_3$ (1 mL) were added to the same volume of treated juice, and the absorbance at 397 nm were measured after incubation for 10 min. Calibration was constructed with quercetin as the standard.

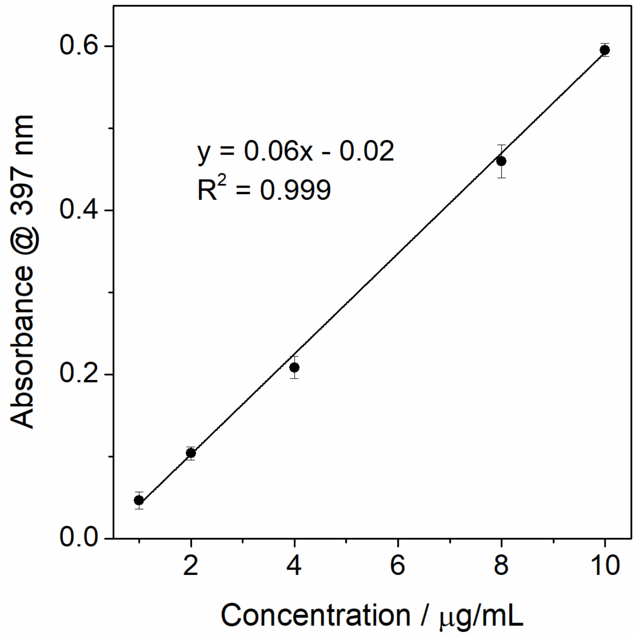

Supplementary Figure 72. The standard curve of quercetin. Error bars = Standard Deviation (n=3).

Supplementary Table 9. Loss ratio of ascorbic acid, phenolic, and flavonoid after different operations.

Type	Operation	Loss ratio			Ref
		Ascorbic Acid	Phenolic	Flavonoid	
Orange	Thermal treatment	23.3%	~57%	-	[15]
Orange	Storage for 6 weeks	~43%	~30%	-	[15]
Pears	Concentrate	-	53.1%	46.5%	[16]
This work	Photodynamic sterilization	2.2%	20%	22.4%	

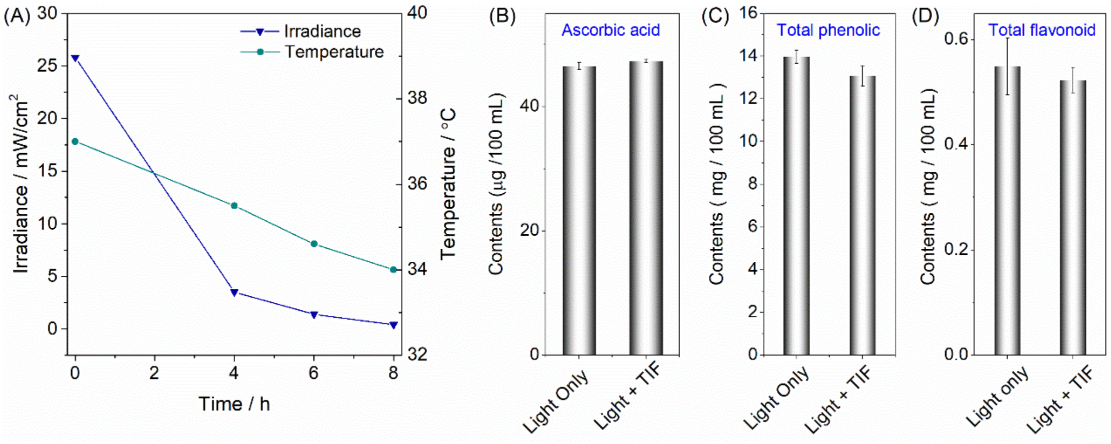

Supplementary Figure 73. Evaluation of the potential influence of sunlight on the contents of the nutrients in passionfruit lemon juice: (A) thee changes of irradiance and temperature of the day that the investigations carried out (10:30-18:30, Aug 14, Chengdu, China); (B) the contents of ascorbic acid under sunlight for 8 hours; (C) the contents of total flavonoid under sunlight for 8 hours; and (D) the contents of total phenolic under sunlight for 8 hours. Error bars = Standard Deviation (n=3).

S9.6 Photodynamic antibacterial with TIF for fresh Fruit preservation

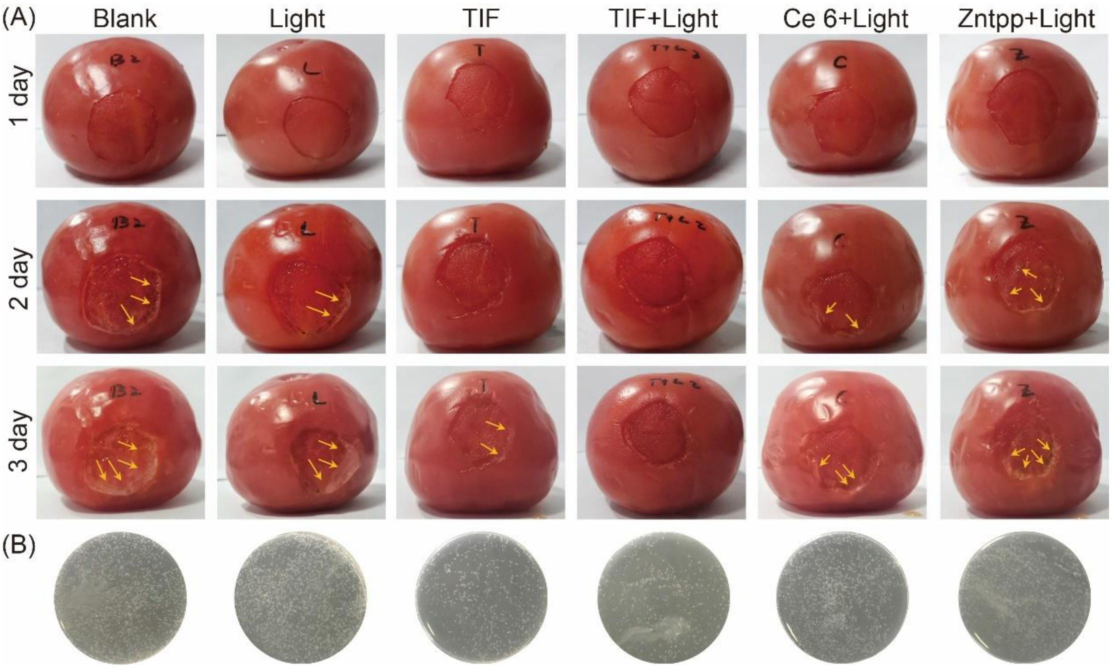

Supplementary Figure 74. Fruit preservation model of TIF. (A) Photographs of tomatoes with different treatments during 1-3 days. (B) Colony counting of infected tomatoes in 3 day.

Section S10. In vivo photodynamic antimicrobial chemotherapy of oral Candidiasis.

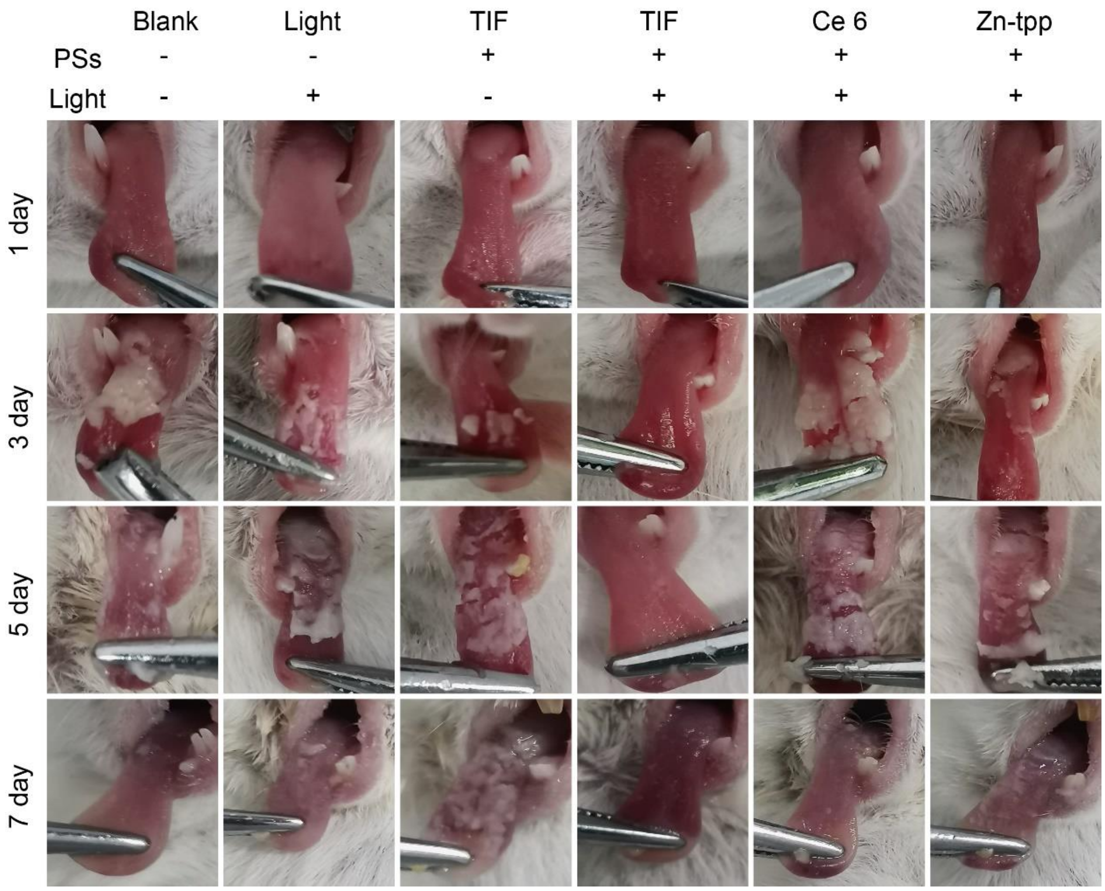

Supplementary Figure 75. Magnified photographs of the oral mucosal infection on the tongue of mice. Error bars = Standard Deviation (n=6).

Supplementary Table 10. The standard of pathology index.[17]

Pathological characteristic	Index
Normal	0
Thin pseudo-membrane, covering the tongue surface area <20%	1
Thin pseudo-membrane, covering the tongue surface area <90% but >20%	2
Thin pseudo-membrane, covering the tongue surface area >91%	3
Thick pseudo-membrane, covering the tongue surface area >91%	4

Supplementary References

1. Wang Y, Chen H, Li C, Wu P. Octachloro-fluorescein: Synthesis and photosensitizer performance evaluation. *Dyes. Pigments.* **170**, 107635 (2019).
2. Sjöback R, Nygren J, Kubista M. Absorption and fluorescence properties of fluorescein. *Spectrochim. Acta. A* **51**, L7-L21 (1995).
3. Lu T, Chen F. Multiwfn: A multifunctional wavefunction analyzer. *J. Comput. Chem.* **33**, 580-592 (2012).
4. Lu T, Chen F. Quantitative analysis of molecular surface based on improved Marching Tetrahedra algorithm. *J. Mol. Graph. Model.* **38**, 314-323 (2012).
5. Lakowicz JR. *Principles of Fluorescence Spectroscopy*, 3rd edn. Springer (2006).
6. Frimannsson DO, Grossi M, Murtagh J, Paradisi F, O'Shea DF. Light Induced Antimicrobial Properties of a Brominated Boron Difluoride (BF2) Chelated Tetraarylazadipyrromethene Photosensitizer. *J. Med. Chem.* **53**, 7337-7343 (2010).
7. Caruso E, Banfi S, Barbieri P, Leva B, Orlandi VT. Synthesis and antibacterial activity of novel cationic BODIPY photosensitizers. *J. Photochem. photobio.B* **114**, 44-51 (2012).
8. Zolfaghari PS*, et al.* In vivo killing of Staphylococcus aureus using a light-activated antimicrobial agent. *BMC Microbiol.* **9**, 27-34 (2009).
9. Lei W, Zhou Q, Jiang G, Zhang B, Wang X. Photodynamic inactivation of Escherichia coli by Ru(ii) complexes. *Photochem. Photobiol. Sci.* **10**, 887-890 (2011).
10. Shao Q, Xing B. Enzyme responsive luminescent ruthenium(ii) cephalosporin probe for intracellular imaging and photoinactivation of antibiotics resistant bacteria. *Chem. Commun.* **48**, 1739-1741 (2012).
11. Liu C*, et al.* Rapid water disinfection using vertically aligned MoS2 nanofilms and visible light. *Nat. Nanotechnol.* **11**, 1098-1104 (2016).
12. Zhang J*, et al.* Phosphorescent Carbon Dots for Highly Efficient Oxygen Photosensitization and as Photo-oxidative Nanozymes. *ACS Appl. Mater. Interfaces* **10**, 40808-40814 (2018).
13. Li P*, et al.* Metal-organic frameworks with photocatalytic bactericidal activity for integrated air cleaning. *Nat. Commun.* **10**, 2177-2186 (2019).
14. Ghafar MFA, Prasad KN, Weng KK, Ismail A. Flavonoid, hesperidine, total phenolic contents and antioxidant activities from Citrus species. *Afr. J. Biotechnol.* **9**, 326-330 (2010).
15. Khandpur P, Gogate PR. Effect of novel ultrasound based processing on the nutrition quality of different fruit and vegetable juices. *Ultrason. Sonochem.* **27**, 125-136 (2015).
16. Jiang G-H, Nam S-H, Yim S-H, Kim Y-M, Gwak HJ, Eun J-B. Changes in total phenolic and flavonoid content and antioxidative activities during production of juice concentrate from Asian pears (Pyrus pyrifolia Nakai). *Food Sci. Biotechnol.* **25**, 47-51 (2016).
17. Hayama K*, et al.* A d-octapeptide drug efflux pump inhibitor acts synergistically with azoles in a murine oral candidiasis infection model. *FEMS Microbiol. Lett.* **328**, 130-137 (2012).
